# Supplementary material for: Plasma type I collagen α1 chain in relation to coronary artery disease: findings from a prospective population-based cohort and an acute myocardial infarction prospective cohort in Sweden
Source: BMJ Open. 2023 Sep 15;13(9):e073561. doi: 10.1136/bmjopen-2023-073561 (PMC10510861; doi:10.1136/bmjopen-2023-073561)
Supplement: Supplementary data [file bmjopen-2023-073561supp001.pdf]

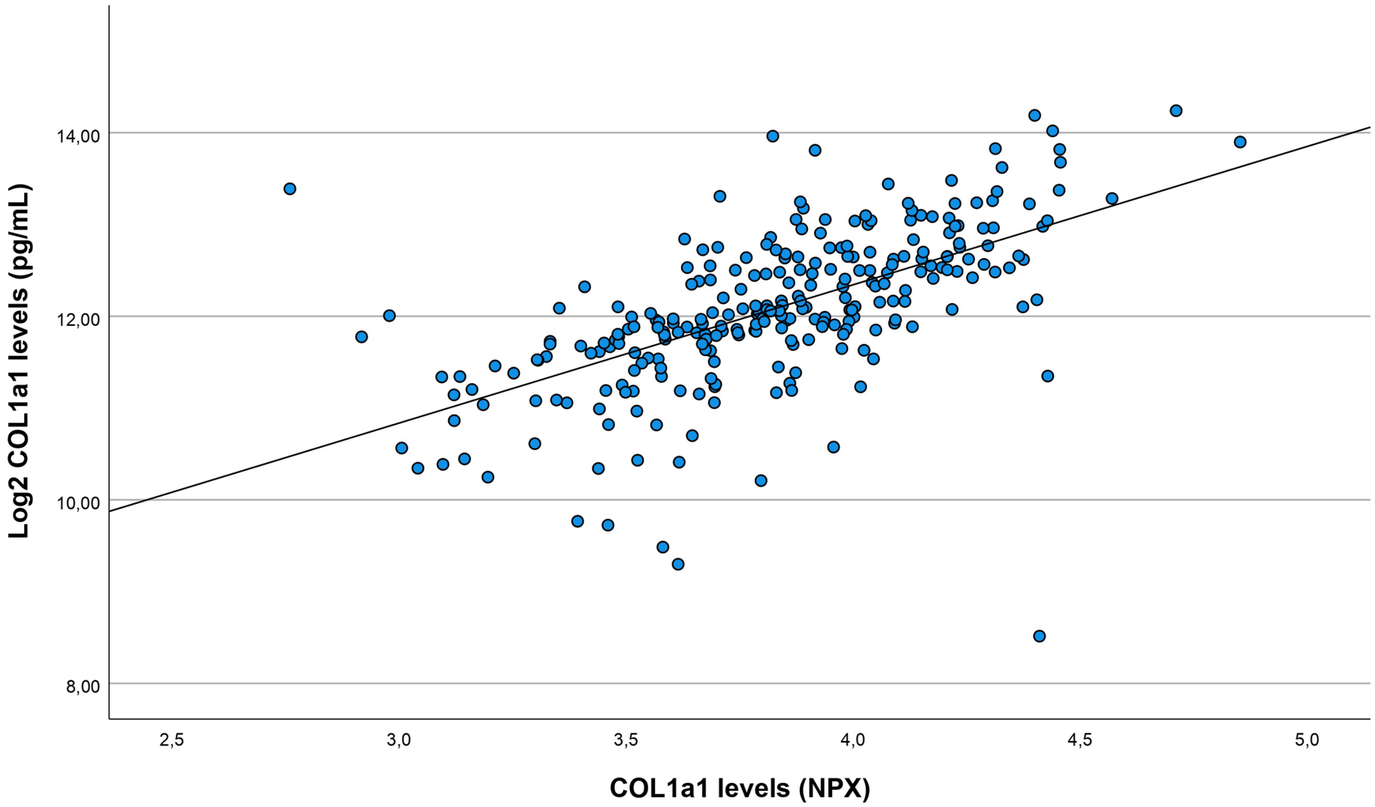

Supplementary figure 1. **Scatter plot showing COL1α1 plasma levels in LSH participants based on Luminex-analysis (y-axis, $\log_2$) versus PEA-analysis (x-axis) along with linear regression line (COL1α1 = collagen type I α1 chain; Log2 = logarithmized with the base 2; NPX = normalized protein expression)**
