# Supplementary material for: Plasma type I collagen α1 chain in relation to coronary artery disease: findings from a prospective population-based cohort and an acute myocardial infarction prospective cohort in Sweden
Source: BMJ Open. 2023 Sep 15;13(9):e073561. doi: 10.1136/bmjopen-2023-073561 (PMC10510861; doi:10.1136/bmjopen-2023-073561)
Supplement: Supplementary data [file bmjopen-2023-073561supp002.pdf]

**Supplementary table 1.** Correlations between COL1α1, PRO-C1, C1M, demographic, clinical and laboratory variables in the LSH cohort expressed as correlation coefficients (*r*) and *p*-values

| Variable | COL1α1 | | PRO-C1 | | C1M | |
|---|---|---|---|---|---|---|
| | *r* | *p* | *r* | *p* | *r* | *p* |
| Age | -0.01 | *ns* | -0.08 | *ns* | 0.13 | 0.035 |
| Sex† | 0.19 | 0.002 | 0.21 | 0.001 | 0.13 | 0.045 |
| BMI | -0.03 | *ns* | -0.16 | 0.015 | 0.15 | 0.015 |
| Diabetes† | -0.15 | 0.014 | -0.09 | *ns* | 0.13 | 0.04 |
| Angina pectoris† | -0.18 | 0.003 | -0.17 | 0.007 | 0.08 | *ns* |
| HDL cholesterol | 0.13 | 0.029 | 0.19 | 0.003 | -0.02 | *ns* |
| Non-HDL cholesterol | 0.07 | *ns* | -0.04 | *ns* | 0.13 | 0.041 |
| Triglycerides | -0.08 | *ns* | -0.17 | 0.007 | 0.14 | 0.033 |
| CRP | 0.06 | *ns* | 0.01 | *ns* | 0.30 | <0.001 |
| IL-6 | -0.05 | *ns* | -0.16 | 0.017 | 0.21 | 0.001 |
| MMP-9 | -0.10 | *ns* | -0.20 | 0.002 | 0.07 | *ns* |
| Pro-C1 | 0.73 | <0.001 | - | - | -0.03 | *ns* |
| C1M | <0.01 | *ns* | -0.04 | *ns* | - | - |

COL1α1 = collagen type I α1 chain; BMI = body mass index; HDL = high density lipoprotein; CRP = C-reactive protein; IL-6 = interleukin-6; MMP-9 = matrix metalloproteinase-9; *ns* = non-significant
Correlations calculated with Spearman analysis. $p < 0.05$ considered statistically significant
† Male coded as 1 and female as 2, diabetes coded as 2 and not having diabetes coded as 1, angina pectoris coded as 2 and not having angina pectoris coded as 1
