# Supplementary material for: Plasma type I collagen α1 chain in relation to coronary artery disease: findings from a prospective population-based cohort and an acute myocardial infarction prospective cohort in Sweden
Source: BMJ Open. 2023 Sep 15;13(9):e073561. doi: 10.1136/bmjopen-2023-073561 (PMC10510861; doi:10.1136/bmjopen-2023-073561)
Supplement: Supplementary data [file bmjopen-2023-073561supp003.pdf]

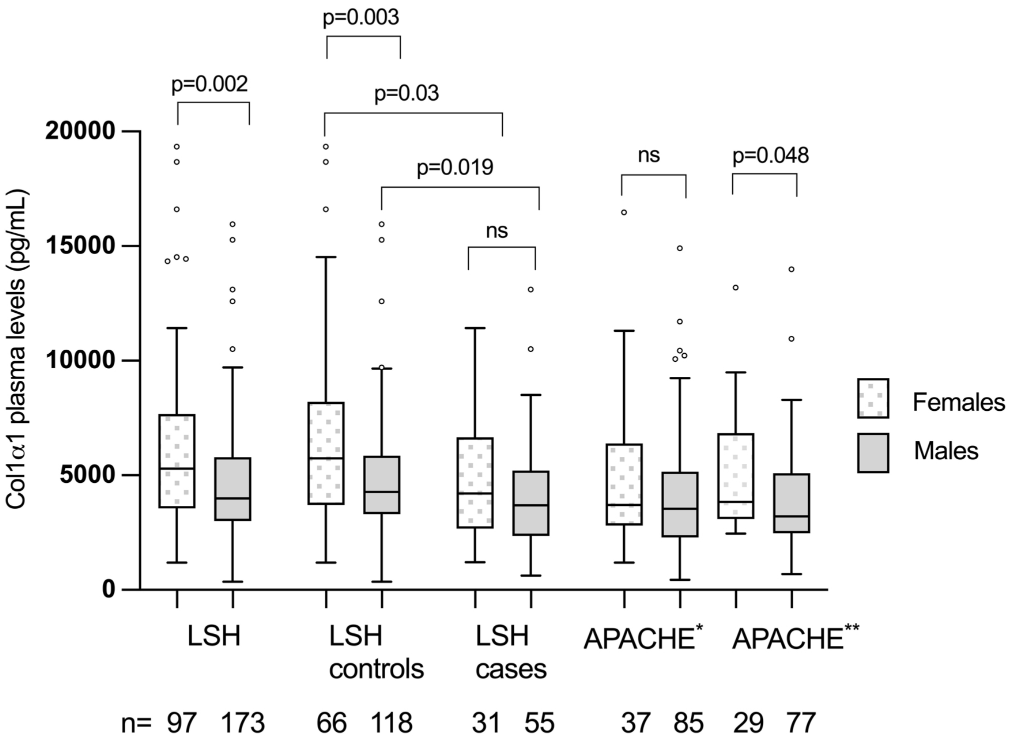

Supplementary figure 2. Box plots showing COL1α1 plasma levels (pg/mL) measured by the Luminex® Assay in LSH and APACHE cohorts split on sex. ***P*-values based upon Mann-Whitney U tests (APACHE = Assessing Platelet Activity in Coronary Heart Disease; COL1α1 = collagen type I α1 chain; LSH = Lifeconditions, Stress and Health study)**

***At admission**
**** 6 months after admission**
