# Supplementary material for: Plasma type I collagen α1 chain in relation to coronary artery disease: findings from a prospective population-based cohort and an acute myocardial infarction prospective cohort in Sweden
Source: BMJ Open. 2023 Sep 15;13(9):e073561. doi: 10.1136/bmjopen-2023-073561 (PMC10510861; doi:10.1136/bmjopen-2023-073561)
Supplement: Author's manuscript [file bmjopen-2023-073561.draft_revisions.pdf]

BMJ Open is committed to open peer review. As part of this commitment we make the peer review history of every article we publish publicly available.

When an article is published we post the peer reviewers' comments and the authors' responses online. We also post the versions of the paper that were used during peer review. These are the versions that the peer review comments apply to.

The versions of the paper that follow are the versions that were submitted during the peer review process. They are not the versions of record or the final published versions. They should not be cited or distributed as the published version of this manuscript.

BMJ Open is an open access journal and the full, final, typeset and author-corrected version of record of the manuscript is available on our site with no access controls, subscription charges or pay-per-view fees (http://bmjopen.bmj.com).

If you have any questions on BMJ Open's open peer review process please email info.bmjopen@bmj.com

BMJ Open

Plasma type I collagen α1 chain in relation to coronary artery disease: findings from a prospective population-based cohort and an acute myocardial infarction cohort in Sweden

Journal:	*BMJ Open*
Manuscript ID	bmjopen-2023-073561
Article Type:	Original research
Date Submitted by the Author:	09-Mar-2023

Complete List of Authors:	Hammaréus, Filip; Linkoping University Department of Health Medicine and Caring Sciences<br>Nilsson, Lennart; Linkoping University Department of Health Medicine and Caring Sciences<br>Ong, Kwok-Leung; University of New South Wales School of Clinical Medicine<br>Kristenson, Margareta; Linkoping University Department of Health Medicine and Caring Sciences<br>Festin, Karin; Linkoping University Department of Health Medicine and Caring Sciences<br>Lundberg, Anna; Linkoping University Department of Health Medicine and Caring Sciences<br>Chung, Rosanna; Linkoping University Department of Health Medicine and Caring Sciences<br>Swahn, Eva; Linköping University, Department of Health, Medicine and Caring Science<br>Alfredsson, Joakim; Linkoping University Department of Health Medicine and Caring Sciences<br>Holm Nielsen, Signe; Technical University of Denmark Department of Biotechnology and Biomedicine; Nordic Bioscience<br>Jonasson, Lena; Linkoping University Department of Health Medicine and Caring Sciences

Keywords:	Coronary heart disease < CARDIOLOGY, Vascular medicine < INTERNAL MEDICINE, Ischaemic heart disease < CARDIOLOGY

SCHOLARONE™
Manuscripts

I, the Submitting Author has the right to grant and does grant on behalf of all authors of the Work (as defined in the below author licence), an exclusive licence and/or a non-exclusive licence for contributions from authors who are: i) UK Crown employees; ii) where BMJ has agreed a CC-BY licence shall apply, and/or iii) in accordance with the terms applicable for US Federal Government officers or employees acting as part of their official duties; on a worldwide, perpetual, irrevocable, royalty-free basis to BMJ Publishing Group Ltd ("BMJ") its licensees and where the relevant Journal is co-owned by BMJ to the co-owners of the Journal, to publish the Work in this journal and any other BMJ products and to exploit all rights, as set out in our licence.

The Submitting Author accepts and understands that any supply made under these terms is made by BMJ to the Submitting Author unless you are acting as an employee on behalf of your employer or a postgraduate student of an affiliated institution which is paying any applicable article publishing charge ("APC") for Open Access articles. Where the Submitting Author wishes to make the Work available on an Open Access basis (and intends to pay the relevant APC), the terms of reuse of such Open Access shall be governed by a Creative Commons licence – details of these licences and which Creative Commons licence will apply to this Work are set out in our licence referred to above.

Other than as permitted in any relevant BMJ Author's Self Archiving Policies, I confirm this Work has not been accepted for publication elsewhere, is not being considered for publication elsewhere and does not duplicate material already published. I confirm all authors consent to publication of this Work and authorise the granting of this licence.

For peer review only

Plasma type I collagen α1 chain in relation to coronary artery disease: findings from a prospective population-based cohort and an acute myocardial infarction cohort in Sweden

Filip Hammaréus[a], Lennart Nilsson[a], Kwok Leung Ong[b], Margareta Kristenson[c], Karin Festin[c], Anna K Lundberg[d], Rosanna WS Chung[d], Eva Swahn[e], Joakim Alfredsson[e], Signe Holm Nielsen[fg†], Lena Jonasson[e†]

[a] Department of Medicine in Jönköping and department of Health, Medicine and Caring Sciences, Unit of Cardiovascular Medicine, Linköping University, Linköping, Sweden
[b] Lipid Research Group, School of Medical Sciences, University of New South Wales, Sydney, NSW, Australia
[c] Department of Health, Medicine and Caring Sciences, Division of Society and Health, Linköping University, Linköping, Sweden
[d] Department of Health, Medicine and Caring Sciences, Unit of Cardiovascular Medicine, Linköping University, Linköping, Sweden
[e] Department of Cardiology in Linköping, and Department of Health, Medicine and Caring Sciences, Unit of Cardiovascular Medicine, Linköping University, Linköping, Sweden
[f] Biomedicine and Biotechnology, Technical University of Denmark, Lyngby, Denmark
[g] ImmunoScience, Nordic Bioscience, Herlev, Denmark
† Shared authorship

Funding source

The LSH study was funded by the Swedish Research Council (2004−1881) and the Swedish Heart and Lung Foundation (2004053). The research was supported by the County Council of Östergötland, specifically with a grant aimed towards scientists early in their career (RÖ-910951); Academy of Health Care, County Council of Jönköping (Futurum); and The Danish Research Foundation and Innovation Fund Denmark. Further, we acknowledge the support from Linköping university.

Corresponding author:

Lennart Nilsson
+46-73-270 38 44
Lennart.nilsson@liu.se
Länssjukhuset Ryhov, 551 85, Jönköping, Sweden

Word counts main text: 3658.

For peer review only

Abstract

objectives: to confirm an association between type I collagen α1 chain (COL1α1) levels and coronary artery disease (CAD) by using absolute quantification in plasma. Also, to investigate the correlates of COL1α1 to clinical characteristics and circulating markers of collagen metabolism.

design: Lifeconditions, Stress and Health (LSH) study: prospective cohort study with a nested case-control design.
Assessing Platelet Activity in Coronary Heart Disease (APACHE) study: prospective cohort study.

setting: LSH: primary care setting.
APACHE: cardiology department, university hospital.

participants: LSH: 1007 randomly recruited individuals aged 45-69 (50% female), exclusion criteria were serious disease. After 13 years follow-up, 86 cases with first-time major event of CAD, defined as fatal/non-fatal myocardial infarction (MI) or coronary revascularization were identified and sex- and age-matched to 184 controls.
APACHE: 125 MI patients, 73 with ST-elevation MI and 52 with non-ST-elevation MI. Exclusion criteria: Intervention study participation, warfarin treatment, short life expectancy and unwillingness to participate.

primary and secondary outcome measures: Primary outcome was the association between baseline COL1α1 and CAD after 13 years (LSH). Secondary outcomes were the association between the collagen biomarkers PRO-C1/C1M and CAD (LSH); temporal changes of COL1α1 after acute MI up to 6 months (APACHE) and lastly, correlates between COL1α1 and patient characteristics, circulating markers of collagen metabolism (LSH and APACHE).

results: COL1α1 levels were associated with CAD, both unadjusted (HR=0.69, 95% CI=0.56-0.87) and adjusted (HR=0.55, 95% CI=0.41-0.75). PRO-C1 was associated with CAD, unadjusted (HR=0.62, 95% CI=0.47-0.82) and adjusted (HR=0.61, 95% CI=0.43-0.86), while C1M was not. In MI patients, COL1α1 remained unchanged up to 6 months. COL1α1 was correlated to PRO-C1, but not to C1M.

conclusions: In conclusion, plasma COL1α1 was independently and inversely associated with CAD. Furthermore, COL1α1 appeared to reflect collagen synthesis but not degradation. Future studies are needed to confirm whether COL1α1 is a clinically useful biomarker of CAD.

For peer review only

Keywords

Atherosclerosis, Coronary artery disease, Extracellular matrix, Collagen

For peer review only - http://bmjopen.bmj.com/site/about/guidelines.xhtml

Strengths and limitations of this study

- The study includes two well-characterized cohorts that complement each other in elucidating the role of type I collagen α1 chain (COL1α1) in coronary artery disease.
- We used absolute quantification of COL1α1 in plasma, as compared to a previous study using a relative quantification method.
- We also quantified biomarkers reflecting collagen synthesis and degradation, respectively, along with markers of inflammation and myocardial injury.
- Although we adjusted for the most common cardiovascular risk factors in our models, there might be residual confounding factors that explain the observed associations.
- The study design and limited sample size (especially women) makes it hard to draw any definitive mechanistic conclusions from our results.

For peer review only

For peer review only - http://bmjopen.bmj.com/site/about/guidelines.xhtml

Introduction

Cardiovascular disease is the leading cause of death and disease burden worldwide. Most clinical manifestations, such as coronary artery disease (CAD), are caused by atherosclerosis (1). Atherosclerosis is a complex and long-lasting process in which multiple factors such as lipid profile, inflammation and extracellular matrix (ECM) remodeling contribute to the phenotype of the atherosclerotic plaque. Whether a plaque develops into a vulnerable one, with increased risk of atherothrombotic complications, largely depends on its phenotype (2). The ECM remodeling process in the arterial wall involves the synthesis and breakdown of ECM proteins such as collagens, proteoglycans and elastin (3). Excessive or uncontrolled ECM remodeling in CAD has been proposed by previous studies showing that ECM-degrading enzymes, such as matrix metalloproteinases (MMPs), are upregulated in rupture-prone plaques (4-6). Also, circulating levels of MMPs, MMP-9 in particular, have been shown to predict the risk of CAD (7, 8). Type I collagen is the most abundant protein in the human body and expressed in numerous tissues including skin, bone, tendons and arteries. It consists of two identical polypeptide α1 chains (COL1α1) and one α2 chain making up a triple helix molecule. The precursor to mature collagen in tissues, pro-collagen, has three major domains: an N-terminal pro-collagen domain, a central triple helical domain and a C-terminal pro-collagen domain (9). The N- and C-terminal pro-collagens, which are cleaved off the central domain, reflect synthesis of type I collagen when measured in plasma (10). Breakdown fragments of the triple helical domain on the other hand, reflect degradation of type I collagen (11). In atherosclerotic plaques, type I collagen comprises 60% of the total protein content and is the primary component of the fibrous cap (12, 13). The type I collagen content is associated with a stable plaque phenotype. Conversely, reduced synthesis and/or increased degradation of type I collagen may lead to thinning of the fibrous cap and thereby increase the risk of plaque rupture (5, 14, 15). In line with this, circulating markers of collagen type I degradation have been shown to predict incident CAD (16, 17). Whether biomarkers reflecting collagen type I synthesis can be used to predict cardiovascular risk has been studied to a lesser extent. Recently, we performed an exploratory study using relative quantification of 184 cardiovascular disease-related proteins to identify plasma biomarkers that predicted CAD over a 13-year follow-up period in a population-based Swedish cohort (18). Interestingly, αα1 was significantly and inversely associated with the risk of incident CAD, defined as first time fatal/non-fatal myocardial infarction (MI) and/or an event of invasive coronary revascularization. It also appeared to be the only biomarker which remained significant after multivariate adjustments for common cardiovascular risk factors. Here, the overall aim was to further investigate COL1α1 in plasma as a novel biomarker in CAD. First, we assessed the association between COL1α1 and future risk of CAD by using absolute quantification in plasma. Secondly, we assessed whether PRO-C1 and C1M, two biomarkers reflecting type I collagen synthesis and degradation, respectively, were associated with CAD in the same cohort. Thirdly, COL1α1 was quantified in a time-course study on patients with acute MI from admission to 6 months after discharge. In both cohorts, we investigated the correlates of COL1α1, including a number of demographic, clinical and laboratory variables.

For peer review only

For peer review only - http://bmjopen.bmj.com/site/about/guidelines.xhtml

Methods

Study population I: The LSH cohort

The longitudinal Life conditions, Stress and Health (LSH) study has been described previously (8, 18). In summary, the LSH cohort consisted of 1007 individuals, aged 45-69 years and evenly distributed by gender, that were randomly recruited from 10 healthcare centers in the County of Östergötland in Southeast Sweden. The participation rate was 62.5%. Exclusion criteria were serious physical or psychiatric disease. Data were collected from the end of 2003 to the middle of 2004, using questionnaires and a visit to their primary healthcare center for health status measurements, including blood pressure and collection of fasting venous samples. The measurements of C-reactive protein (CRP), interleukin (IL)-6 and MMP-9 in plasma have been described previously (8). The primary outcome measure during 13 years of follow-up was onset of first-time major event of CAD, defined as fatal/non-fatal MI or coronary revascularization (percutaneous coronary intervention or coronary artery bypass surgery). Outcome data was collected from the Cause of Death Registry and the Registry of Hospital Admissions (Swedish National Board of Health and Welfare). In total, 86 cases with a primary outcome were identified, and for a nested case control design 184 age- and sex-matched controls were identified.

Study population II: The APACHE cohort

The study of Assessing Platelet Activity in Coronary Heart Disease (APACHE) has been described previously (19). The APACHE cohort consisted of 125 MI patients, whereof 73 with ST-elevation MI (STEMI) and 52 with non-ST-elevation MI (NSTEMI), defined according to the global definition of MI (20). The patients were recruited between January 2009 and August 2011 at the University Hospital Linköping, Sweden. Exclusion criteria were that the patient: already participated in an intervention study, were treated with warfarin before admission, had short life expectancy (less than 6 months) and were unwilling to participate. Coronary angiography was performed in all STEMI patients immediately after admission to hospital and in 98 % of NSTEMI patients within 72 hours. Left ventricular function was assessed by echocardiography 2-3 days after admission. Patients were classified as having normal, mildly reduced, moderately reduced or severely reduced ventricular function according to established American/European guidelines (21). Blood samples were collected at 4 time points: at admission (n=122), 3 days (n=117), 7-9 days (n=110) and 6 months after admission (n=106). Laboratory tests such as lipid profile and CRP were analyzed according to clinical routine between day 2-4 after admission. High sensitivity Troponin T (hsTnT) was assessed 6-8 hours after admission. MMP-9 and IL-6 were analyzed according to manufacturer´s instructions in plasma from admission using commercially available ELISA kits (R&D Systems, Minneapolis MN, USA).

Quantification of COL1α1 in plasma

In the quantification of COL1α1 in plasma, all samples were sodium heparin plasma, except 15 samples in the LSH cohort that were EDTA-plasma (3 cases, 12 controls). Plasma was stored in -70°C until analysis. After thawing, the sample tubes were centrifuged at 16 000 x g for 4 minutes right before analysis. A Magnetic Luminex® Assay (R&D Systems Inc., Minneapolis, MN, USA, Catalog Number LXSAHM) was used to quantify COL1α1 according to manufacturer's instructions. The Magnetic Luminex® Assay steps and principles have been described in detail elsewhere (22). An automatic plate washer, HydroFlex™ (Tecan Trading AG, Switzerland), was used for washing steps. The samples were analyzed on a Flexmap 3D analyzer (Luminex corp., Hertogenbosch, Netherlands) platform using the Bioplex manager software (Biorad) to create a standard curve with a five-parameter logistic curve fit. On every assay plate, with 96 wells per plate, a duplicate of control plasma was

For peer review only - http://bmjopen.bmj.com/site/about/guidelines.xhtml

analyzed to calculate intra- and inter-assay coefficients of variation (CV). In the LSH samples, the intra-assay CV ranged from 0.4% to 7.0% with an inter-assay CV of 14.6%. In the APACHE samples, intra-assay CV ranged from <0.1% to 4.4% and inter-assay CV amounted to 21.1%. Lower limit of quantification (LLOQ) ranged from 735-750 pg/mL while upper limit of quantification (ULOQ) ranged between 175 475-176 100 pg/mL. Three samples in LSH (2 controls, 1 case) were below the LLOQ and excluded from analysis. The type of anticoagulant did not seem to affect plasma levels of COL1α1, e.g., in LSH controls, the levels in sodium heparin plasma (n=172) did not differ significantly from those in EDTA-plasma (n=12), median 4410 pg/mL *vs.* 4490 pg/mL ($p = 0.21$).

Proximity extension assay (PEA) measurements of COL1α1 in the LSH study have been described previously (18). In short, 184 cardiovascular disease-related proteins, one of which was COL1α1, were measured in 270 LSH plasma samples. The PEA-technique utilizes DNA oligonucleotide labeled antibody probes to quantify protein concentrations through real-time polymerase chain reaction technology. The protein concentrations are presented as normalized protein expression (NPX) values. Higher and lower NPX values corresponds to higher and lower protein concentrations, respectively, although not being absolute concentration values.

Quantification of PRO-C1 and C1M in plasma

Two neo-epitope specific biomarkers targeting type I collagen formation (PRO-C1) and type I collagen degradation (C1M) were measured in 255 heparin plasma samples (83 cases and 172 controls) in the LSH cohort using competitive ELISAs developed by Nordic Bioscience (Herlev, Denmark) (10, 11).The inter- and intra-assay coefficients of variation were <15% and <10% respectively for both assays. In the case of PRO-C1, 3 samples were below LLOQ while C1M had 21 samples below LLOQ along with 1 sample above ULOQ, we excluded these samples from further analysis.

Statistical analysis

Descriptive statistics were used to yield means, medians, standard deviations (SDs) and interquartile ranges (IQR). Medians (IQR) were presented instead of mean (SD) if the variable did not follow distribution of normality in any of the groups presented. Kolmogorov-Smirnov test was used for testing the distribution of normality for the variables. For comparison between two groups, independent samples t-test was used for normally distributed variables and Mann Whitney U-test for non-normally distributed variables. Correlation analyses were performed using Spearman´s analysis. A multiple linear regression model was used when testing for independent predictors of biomarker levels. Variables significantly correlated to biomarker levels in Spearman's analysis were included as non-dependent variables in these models. In APACHE, Friedman analysis was used to explore if there was a significant difference in COL1α1 level across the four time points. To investigate the hazard ratio (HR) for COL1α1, pro-C1 and C1M in terms of primary outcome (see definition above) in the LSH population, a Cox proportional hazard regression analysis was performed, both with and without covariates. In accordance with our previous study (18), covariates being adjusted for were established cardiovascular risk factors: sex, age, systolic blood pressure, smoking, body mass index, triglycerides, high-density lipoprotein (HDL) cholesterol, non-HDL cholesterol, CRP, use of anti-hypertensive and lipid-lowering medication, and presence of chronic disease. When performing the Cox proportional hazard analysis, we used the $\log_2$ value of COL1α1. Pro-C1 and C1M Cox proportional hazard analyses were performed with $\log_2$ values as well. When assessing the difference in cumulative survival in terms of our primary outcome between groups the log rank test was performed to test for statistical significance along with plotting a Kaplan Meier curve for visualization. Two-tailed $p < 0.05$

For peer review only - http://bmjopen.bmj.com/site/about/guidelines.xhtml

was used as cut off for statistical significance. IBM SPSS, release 28 (IBM Corporation, Armonk, New York) was used for all statistical analyses.

Ethical considerations

As previously described (8, 19), the LSH and APACHE studies have been approved by The Regional Ethical Review Board in Linköping (Dnr 02–0324 and M45-08 respectively). All study participants have given written informed consent.

Patient and Public Involvement statement

At the time of conducting this research, patients and/or the public were not involved in the design, conduct, reporting or dissemination of the research.

For peer review only

For peer review only - http://bmjopen.bmj.com/site/about/guidelines.xhtml

Results

Plasma levels of COL1α1 and their association with incident CAD in the LSH cohort

Demographic, clinical and laboratory characteristics of LSH cases (n=86) and controls (n=184) are presented in **Table 1**. The mean age was 61 years and around 64 % were men. The cases smoked more, had higher blood pressures, higher levels of non-HDL cholesterol, triglycerides, CRP and MMP-9 and lower levels of HDL cholesterol compared with controls. As shown in **Table 2**, the absolute concentrations of COL1α1 measured by Luminex were significantly lower in LSH cases compared with controls. Furthermore, the absolute concentrations of COL1α1 exhibited a significant correlation with the PEA-based relative concentrations of COL1α1 ($r = 0.69$, $p < 0.001$, see **Supplementary figure 1)**. The PEA-based data were previously published by Ong *et al.* (18).

We next proceeded to assess whether absolute concentrations of COL1α1 were associated with incident CAD at 13 years follow-up (**Table 2**). When performing Cox proportional hazard analysis, COL1α1 levels showed a significant association with incident CAD in the unadjusted model (HR = 0.69, 95% CI = 0.56-0.87, $p = 0.001$). In the adjusted model, COL1α1 levels remained significantly associated with incident CAD (HR = 0.55, 95% CI = 0.41-0.75, $p < 0.001$). Furthermore, when the LSH participants were separated into two groups based on COL1α1 values above or under median (≥ or < 4010 pg/mL), the cumulative survival in terms of CAD outcome differed significantly ($p = 0.002$, **Figure 1a**).

Plasma levels of PRO-C1 and C1M and their associations with incident CAD in the LSH cohort

As further shown in **Table 2**, PRO-C1 levels were significantly lower in cases compared with controls in LSH, while C1M levels did not differ significantly between the groups. PRO-C1 levels showed a significant association with CAD outcome both in the unadjusted (HR = 0.62, 95% CI = 0.47 – 0.82, $p = 0.001$) and adjusted model (HR = 0.61, 95% CI = 0.43 – 0.86, $p = 0.005$). On the other hand, C1M levels did not show any significant association with CAD outcome. Cumulative survival differed significantly in terms of CAD outcome when the LSH cohort was split based on PRO-C1 levels above or under median (≥ or < 71.9 ng/mL) ($p < 0.001$, **Figure 1b**).

Temporal changes of plasma COL1α1 in the APACHE cohort

Demographic, clinical and laboratory characteristics of the APACHE cohort, 73 patients with STEMI and 52 patients with NSTEMI, are shown in **Table 3**. The mean age was 66 years and around 70% were men. The COL1α1 levels did not change significantly from admission to 6 months, neither in STEMI nor in NSTEMI patients. In terms of different time points, COL1α1 median (IQR) value in the APACHE cohort was 3590 (3200), 3780 (3100), 3570 (3300) and 3330 (2800) pg/mL at arrival, after 3 days, after 7-9 days and after 6 months, respectively ($p = 0.92$). As shown in **Figure 2**, COL1α1 levels in the APACHE cohort (at admission) were comparable to COL1α1 levels in the LSH cases.

Correlates of COL1α1, PRO-C1 and C1M

Among baseline characteristics in the LSH cohort, COL1α1 showed correlations with female sex, HDL cholesterol, diabetes and angina pectoris (**Supplementary table 1)**. In a multiple regression model, only female sex remained significantly associated with COL1α1 levels (*beta* = 0.21, $p = 0.001$). As further shown in **Supplementary table 1**, PRO-C1 correlated with female sex, BMI, angina pectoris, triglycerides, HDL cholesterol, IL-6 and MMP-9. In a multiple regression model, only female sex remained significantly associated with PRO-C1 levels (*beta* = 0.23, $p = 0.001$). Further, C1M correlated to female sex, age, BMI, triglycerides, non-HDL cholesterol, CRP, diabetes and IL-6. However, in a multiple regression model, only CRP (*beta* = 1.68, $p < 0.001$) and non-HDL cholesterol

For peer review only - http://bmjopen.bmj.com/site/about/guidelines.xhtml

(*beta* = 1.80, $p = 0.032$) remained significantly related to C1M levels. When examining the intercorrelations between COL1α1, PRO-C1 and C1M in the LSH cohort, PRO-C1 and COL1α1 showed a strong correlation ($r = 0.73$, $p < 0.001$) while C1M did not correlate to either COL1α1 or PRO-C1.

In the APACHE cohort, female sex was the only variable that showed a significant correlation with COL1α1 concentrations in bivariate correlation analyses, though only at 6 months ($r = 0.19$, $p = 0.047$). COL1α1 did not showcase any significant correlations to IL-6, MMP-9 or CRP in LSH nor in APACHE. Neither was it correlated to hsTnT or to left ventricular dysfunction in APACHE.

As shown in **Supplementary Figure 2**, women had significantly higher COL1α1 levels than men in the LSH cohort. Also, female controls had significantly higher COL1α1 levels than female cases. Furthermore, PRO-C1 levels were higher in women than in men, 85.6 (60) *vs* 67.3 (32) ng/mL ($p = 0.001$) while C1M levels did not differ between the sexes (data not shown). In the APACHE cohort, the difference in COL1α1 levels at admission between men and women did not reach significance but was significantly different at 6 months, median (IQR) 3200 (2600) *vs.* 3840 (3700) pg/mL, respectively ($p = 0.048$).

Discussion

The main finding of the present study was that absolute concentrations of plasma COL1α1 levels were independently and inversely associated with incident CAD after 13 years in a population-based cohort. PRO-C1 showed a similar relationship to CAD outcome while C1M did not. Further investigation in a MI population showed that COL1α1 levels did not change significantly during a 6-month period after the acute event.

In the population-based cohort, COL1α1 levels in plasma were significantly lower in those who developed CAD after 13 years of follow-up compared with those who did not. A cox proportional hazard analysis confirmed the association between lower COL1α1 levels and CAD outcome, even after multiple adjustment. The results confirm, and extend, the previously published findings by Ong *et al.* (18). However, in that explorative study, the measurements of COL1α1 were based on relative quantification and it was therefore important to validate the results by a quantifying assay. Prior to the study by Ong *et al.* (18), there were no studies available on COL1α1 in a CAD setting, except for one study reporting that a polymorphism in the COL1α1 gene occurred more frequently in patients surviving a MI, as compared with age-matched controls (23).

Similar to COL1α1, PRO-C1, a biomarker of type I collagen synthesis, was inversely associated with CAD in both unadjusted and adjusted models while C1M, a biomarker for type I collagen breakdown, did not show any association. In line with this, COL1α1 showed a strong correlation with PRO-C1 indicating that both are markers of type I collagen synthesis. COL1α1 is the α1 chain partly making up the type I collagen molecule, whilst PRO-C1 is the internal epitope in the N-terminal pro-peptide of type I collagen (PINP). In other words, they can be regarded as products from two different stages of type I collagen synthesis (9, 10). As far as we know, no previous studies have investigated PRO-C1 in the context of CAD. C1M has, on the other hand, been associated with increased risk of cardiovascular events. One previous study reported that elevated C1M levels predicted MI in a prospective cohort of postmenopausal women (n=5450, median age 71 years) with a median follow-up time of 14 years (17). Another study with a follow-up of 6 years reported that elevated C1M levels predicted cardiovascular events and mortality in patients who had undergone carotid endarterectomy (n = 473, median age 72 years, 64% men) (16). The discrepancy in results between these previous studies and this present study as regards C1M could be due to several reasons. First, the two previous studies measured C1M in serum while we utilized plasma, a distinction that can

For peer review only - http://bmjopen.bmj.com/site/about/guidelines.xhtml

lead to different outcomes with examples from other biomarkers such as MMPs (24). Moreover, the study populations are not comparable in terms of size and composition. For example, the study by Holm Nielsen *et al.* (16) included patients who had undergone carotid endarterectomy yielding an older sample with high atherosclerotic burden compared with our population-based sample.

In the present study, we also investigated the temporal pattern of COL1α1 levels in MI patients by performing serial measurements from admission up to 6 months after the acute event. COL1α1 did not show any temporal changes over time in STEMI or NSTEMI patients, neither did it show any correlation with inflammatory markers or hsTnT in the early phase. Obviously, COL1α1 levels in plasma are not associated with acute atherothrombotic complications, inflammation or myocardial injury and appears individually stable over time. Interestingly, COL1α1 values in patients with acute MI (APACHE) were found to be equal to the values in individuals who developed incident CAD after a 13-year follow-up (LSH cases) as shown in **Figure 2**. Of note, this comparison should be made with caution since it is based on two widely different cohorts.

Women had significantly higher levels of COL1α1 and PRO-C1 compared with men. Female sex was also the only determinant that remained independently associated with COL1α1 and PRO-C1 in a multiple regression model including baseline variables. Collagen homeostasis has been shown to be affected by estrogen (25). PINP and other bone turnover markers are known to increase in women after menopause while there is little increase in men with age (26). This may at least partly explain why women in the population-based cohort had higher levels of COL1α1 and PRO-C1 than men. The sex difference in COL1α1 was also seen in MI patients although not reaching statistical significance in admission plasma, probably owing to the lack of power in this analysis.

The major limitation of our study is the limited sample size, especially as regards women. Variables that were included in the adjustment models are known to be of major importance for CAD risk. Still, it is possible that we have residual confounding factors. A recent study showed that COL1α1 was upregulated in left ventricular heart tissue from heart failure patients and that plasma levels were increased in those who were candidates for heart transplant (27). However, the MI cohort in our study included only 16 patients with moderate or severe left ventricular dysfunction, which does not permit us to draw conclusions about the relationship between COL1α1 and heart failure.

Based on our results, it can be speculated that plasma levels of COL1α1 and PRO-C1 reflect the ability to form type I collagen in arterial tissue, thereby influencing the development and fate of atherosclerotic plaques and hence future CAD risk. However, bones and tendons are other potential sources of COL1α1 in the circulation. Also, the liver may leak ECM components to the blood stream under pathological conditions (28). Whether COL1α1 itself has a causal role in the development of CAD is unclear. Interestingly, a few previous clinical studies have investigated the effect of collagen supplement therapies on atherosclerosis-related factors. These studies, although small and with short intervention periods, have shown positive effects on LDL cholesterol/HDL cholesterol ratio, toxic advanced glycation end-products, cardio-ankle vascular index and brachial-ankle pulse wave velocity (29, 30).

To summarize, the absolute concentration of COL1α1 in plasma was independently and inversely associated with future risk of CAD. It appears to reflect collagen synthesis, but not collagen degradation, inflammation or myocardial injury. Larger studies are needed to confirm the clinical usefulness of COL1α1 as a marker of coronary atherosclerosis and/or predictor of incident CAD.

For peer review only - http://bmjopen.bmj.com/site/about/guidelines.xhtml

Acknowledgments

We would like to acknowledge Bettina Jung and Sofie Madsen for their technical support. The research was supported by a grant from the County Council of Östergötland, Sweden, aimed towards scientists early in their career; Academy of Health Care, County Council of Jönköping (Futurum); and The Danish Research Foundation and Innovation Fund Denmark.

Competing interests

SHN is full-time employees at Nordic Bioscience A/S. Nordic Bioscience is a privately-owned, small–medium size enterprise (SME) partly focused on the development of biomarkers including PRO-C1 and C1M. No fees, bonuses or other benefits was rewarded for the work described in the manuscript. SHN hold stocks in Nordic Bioscience A/S.

Author contributions

Filip Hammaréus wrote the manuscript; Filip Hammaréus, Anna Lundberg, Lena Jonasson, Margareta Kristenson, Eva Swahn and Joakim Alfredsson participated in study design; Filip Hammaréus, Rosanna WS Chung and Anna Kristina Lundberg prepared plasma samples and performed the Luminex-assay on COL1α1; Signe Holm Nielsen measured C1M and PRO-C1 levels in the LSH cohort; Filip Hammaréus, Karin Festin, Lennart Nilsson and Joakim Alfredsson participated in data analysis; All authors participated in data interpretation and critical revision of the manuscript.

Data availability statement

Data are available on reasonable request.

For peer review only

References

1. Herrington W, Lacey B, Sherliker P, Armitage J, Lewington S. Epidemiology of Atherosclerosis and the Potential to Reduce the Global Burden of Atherothrombotic Disease. Circ Res. 2016;118(4):535-46.
2. Bentzon JF, Otsuka F, Virmani R, Falk E. Mechanisms of plaque formation and rupture. Circ Res. 2014;114(12):1852-66.
3. Rekhter MD. Collagen synthesis in atherosclerosis: too much and not enough. Cardiovasc Res. 1999;41(2):376-84.
4. Galis ZS, Khatri JJ. Matrix metalloproteinases in vascular remodeling and atherogenesis: the good, the bad, and the ugly. Circ Res. 2002;90(3):251-62.
5. Galis ZS, Sukhova GK, Lark MW, Libby P. Increased expression of matrix metalloproteinases and matrix degrading activity in vulnerable regions of human atherosclerotic plaques. J Clin Invest. 1994;94(6):2493-503.
6. Gough PJ, Gomez IG, Wille PT, Raines EW. Macrophage expression of active MMP-9 induces acute plaque disruption in apoE-deficient mice. J Clin Invest. 2006;116(1):59-69.
7. Blankenberg S, Rupprecht HJ, Poirier O, Bickel C, Smieja M, Hafner G, et al. Plasma concentrations and genetic variation of matrix metalloproteinase 9 and prognosis of patients with cardiovascular disease. Circulation. 2003;107(12):1579-85.
8. Garvin P, Jonasson L, Nilsson L, Falk M, Kristenson M. Plasma Matrix Metalloproteinase-9 Levels Predict First-Time Coronary Heart Disease: An 8-Year Follow-Up of a Community-Based Middle Aged Population. PLoS One. 2015;10(9):e0138290.
9. Ricard-Blum S. The collagen family. Cold Spring Harb Perspect Biol. 2011;3(1):a004978.
10. Leeming DJ, Larsen DV, Zhang C, Hi Y, Veidal SS, Nielsen RH, et al. Enzyme-linked immunosorbent serum assays (ELISAs) for rat and human N-terminal pro-peptide of collagen type I (PINP)--assessment of corresponding epitopes. Clin Biochem. 2010;43(15):1249-56.
11. Leeming D, He Y, Veidal S, Nguyen Q, Larsen D, Koizumi M, et al. A novel marker for assessment of liver matrix remodeling: an enzyme-linked immunosorbent assay (ELISA) detecting a MMP generated type I collagen neo-epitope (C1M). Biomarkers. 2011;16(7):616-28.
12. Murata K, Motayama T, Kotake C. Collagen types in various layers of the human aorta and their changes with the atherosclerotic process. Atherosclerosis. 1986;60(3):251-62.
13. Shami A, Gonçalves I, Hultgårdh-Nilsson A. Collagen and related extracellular matrix proteins in atherosclerotic plaque development. Curr Opin Lipidol. 2014;25(5):394-9.
14. Edsfeldt A, Gonçalves I, Grufman H, Nitulescu M, Dunér P, Bengtsson E, et al. Impaired fibrous repair: a possible contributor to atherosclerotic plaque vulnerability in patients with type II diabetes. Arterioscler Thromb Vasc Biol. 2014;34(9):2143-50.
15. Wang J, Geng YJ, Guo B, Klima T, Lal BN, Willerson JT, et al. Near-infrared spectroscopic characterization of human advanced atherosclerotic plaques. J Am Coll Cardiol. 2002;39(8):1305-13.
16. Holm Nielsen S, Tengryd C, Edsfeldt A, Brix S, Genovese F, Bengtsson E, et al. A biomarker of collagen type I degradation is associated with cardiovascular events and mortality in patients with atherosclerosis. J Intern Med. 2019;285(1):118-23.
17. Bertelsen DM, Neergaard JS, Bager CL, Nielsen SH, Secher NH, Svendsen JH, et al. Matrix Metalloproteinase Mediated Type I Collagen Degradation is an Independent

For peer review only - http://bmjopen.bmj.com/site/about/guidelines.xhtml

Predictor of Increased Risk of Acute Myocardial Infarction in Postmenopausal Women. Sci Rep. 2018;8(1):5371.
18. Ong KL, Chung RWS, Hui N, Festin K, Lundberg AK, Rye KA, et al. Usefulness of Certain Protein Biomarkers for Prediction of Coronary Heart Disease. Am J Cardiol. 2020;125(4):542-8.
19. Alfredsson J, Lindahl TL, Gustafsson KM, Janzon M, Jonasson L, Logander E, et al. Large early variation of residual platelet reactivity in Acute Coronary Syndrome patients treated with clopidogrel: results from Assessing Platelet Activity in Coronary Heart Disease (APACHE). Thromb Res. 2015;136(2):335-40.
20. Thygesen K, Alpert JS, White HD, Joint ESCAAHAWHFTFftRoMI. Universal definition of myocardial infarction. J Am Coll Cardiol. 2007;50(22):2173-95.
21. Lang RM, Bierig M, Devereux RB, Flachskampf FA, Foster E, Pellikka PA, et al. Recommendations for chamber quantification. Eur J Echocardiogr. 2006;7(2):79-108.
22. Zhang Y, Li X, Di YP. Fast and Efficient Measurement of Clinical and Biological Samples Using Immunoassay-Based Multiplexing Systems. Methods Mol Biol. 2020;2102:129-47.
23. Speer G, Szenthe P, Kosa JP, Tabak AG, Folhoffer A, Fuszek P, et al. Myocardial infarction is associated with Spl binding site polymorphism of collagen type 1A1 gene. Acta Cardiol. 2006;61(3):321-5.
24. Mannello F. Serum or plasma samples? The "Cinderella" role of blood collection procedures: preanalytical methodological issues influence the release and activity of circulating matrix metalloproteinases and their tissue inhibitors, hampering diagnostic trueness and leading to misinterpretation. Arterioscler Thromb Vasc Biol. 2008;28(4):611-4.
25. McRobb LS, McGrath KCY, Tsatralis T, Liong EC, Tan JTM, Hughes G, et al. Estrogen Receptor Control of Atherosclerotic Calcification and Smooth Muscle Cell Osteogenic Differentiation. Arterioscler Thromb Vasc Biol. 2017;37(6):1127-37.
26. Eastell R, Pigott T, Gossiel F, Naylor KE, Walsh JS, Peel NFA. DIAGNOSIS OF ENDOCRINE DISEASE: Bone turnover markers: are they clinically useful? Eur J Endocrinol. 2018;178(1):R19-r31.
27. Hua X, Wang YY, Jia P, Xiong Q, Hu Y, Chang Y, et al. Multi-level transcriptome sequencing identifies COL1A1 as a candidate marker in human heart failure progression. BMC Med. 2020;18(1):2.
28. Nallagangula KS, Nagaraj SK, Venkataswamy L, Chandrappa M. Liver fibrosis: a compilation on the biomarkers status and their significance during disease progression. Future Sci OA. 2018;4(1):FSO250.
29. Tomosugi N, Yamamoto S, Takeuchi M, Yonekura H, Ishigaki Y, Numata N, et al. Effect of Collagen Tripeptide on Atherosclerosis in Healthy Humans. J Atheroscler Thromb. 2017;24(5):530-8.
30. Igase M, Kohara K, Okada Y, Ochi M, Igase K, Inoue N, et al. A double-blind, placebo-controlled, randomised clinical study of the effect of pork collagen peptide supplementation on atherosclerosis in healthy older individuals. Biosci Biotechnol Biochem. 2018;82(5):893-5.

For peer review only - http://bmjopen.bmj.com/site/about/guidelines.xhtml

Figure legends

Figure 1a. Kaplan Meier survival curve showing cumulative survival in terms of primary outcome (MI or invasive coronary intervention) between participants with high or low COL1α1 values (above or under median) in the LSH cohort (COL1α1 = collagen type I α1 chain; Cum = cumulative)

Figure 1b. Kaplan Meier survival curve showing cumulative survival in terms of primary outcome (MI or invasive coronary intervention) between participants with high or low PRO-C1 values (above or under median) in the LSH cohort (COL1α1 = collagen type I α1 chain; Cum = cumulative)

Figure 2. Box plots showing COL1α1 plasma levels (pg/mL) measured by the Luminex® Assay among participants in LSH and APACHE. *P*-value based on Mann-Whitney U test (APACHE = Assessing Platelet Activity in Coronary Heart Disease; COL1α1 = collagen type I α1 chain; LSH = Lifeconditions, Stress and Health study)

*At admission

Supplementary figure 1. Scatter plot showing COL1α1 plasma levels in LSH participants based on Luminex-analysis (y-axis, log_2) versus PEA-analysis (x-axis) along with linear regression line (COL1α1 = collagen type I α1 chain; Log2 = logarithmized with the base 2; NPX = normalized protein expression)

Supplementary figure 2. Box plots showing COL1α1 plasma levels (pg/mL) measured by the Luminex® Assay in LSH and APACHE cohorts split on sex. *P*-values based upon Mann-Whitney U tests (APACHE = Assessing Platelet Activity in Coronary Heart Disease; COL1α1 = collagen type I α1 chain; LSH = Lifeconditions, Stress and Health study)

*At admission

** 6 months after admission

For peer review only - http://bmjopen.bmj.com/site/about/guidelines.xhtml

Tables

Table 1. Baseline characteristics of cases and controls in the LSH cohort

Characteristics	Controls (n=184)	Cases (n=86)	*p*
Age, years	60.6 (6.4)	60.6 (6.6)	0.84
Women, n (%)	66 (36)	31 (36)	>0.9
Smokers, n (%)	19 (11)	24 (30)	<0.001
BMI, kg/m^2	26.8 (3.6)	27.8 (3.8)	0.048
Systolic blood pressure, mmHg	135 (18)	146 (24)	<0.001
Antihypertensive medication, n (%)	32 (17)	30 (35)	0.002
Lipid-lowering medication, n (%)	9 (4.9)	11 (13)	0.027
Diabetes, n (%)	14 (7.6)	8 (9.3)	0.64
Angina pectoris, n (%)	4 (2.2)	12 (14)	<0.001
Serious chronic illness, n (%)	13 (7.1)	9 (11)	0.34
HDL-cholesterol, mmol/L [a]	1.50 (0.50)	1.40 (0.42)	0.001
LDL-cholesterol, mmol/L	3.54 (0.82)	3.64 (0.90)	0.39
Non-HDL cholesterol, mmol/L	4.11 (0.89)	4.36 (0.91)	0.036
Triglycerides, mmol/L[a]	1.10 (0.66)	1.40 (0.92)	<0.001
CRP, mg/L [a]	0.835 (1.7)	1.56 (2.4)	0.01
IL-6, pg/mL [a]	0.560 (2.25)	1.68 (2.5)	0.097
MMP-9, ng/mL [a]	30.7 (32)	39.5 (31)	0.036

BMI = body mass index; HDL = high density lipoprotein; LDL = low density lipoprotein; CRP = C-reactive protein; IL-6 = interleukin-6; MMP-9 = matrix metalloproteinase-9

Data expressed as mean (SD) or median (IQR)[a] if data do not possess distribution of normality. *P*-values from comparing controls and cases in terms of characteristics.

For peer review only

Table 2. Circulating levels of COL1α1, PRO-C1, C1M and their association with incident coronary artery disease at 13 years of follow-up in the LSH cohort

Biomarker	Median (IQR) level				Unadjusted model		Adjusted model†	
	Control (n=184*)	Case (n=86*)	Difference	*p*	HR (95% CI)	*p*	HR (95% CI)	*p*
COL1α1 (pg/mL)	4430 (2900)	3810 (3400)	16%	0.002	0.69 (0.56-0.87)	0.001	0.55 (0.41-0.75)	<0.001
PRO-C1 (ng/mL)	77.6 (43)	59.7 (39)	30%	<0.001	0.62 (0.47-0.82)	0.001	0.61 (0.43-0.86)	0.005
C1M (ng/mL)	22.0 (16)	23.9 (19)	8.6%	*ns*	1.57 (0.568-4.34)	NS	0.92 (0.29-3.37)	*ns*

COL1α1 = collagen type I α1 chain; PRO-C1 = N-terminal pro-peptide of type I collagen; C1M = MMP-mediated degradation of type I collagen; HR = hazard ratio; *ns* = non-significant

Association expressed as hazard ratios per one unit increase in log2-tranformed unit from Cox proportional hazard analysis

*For pro-C1 and C1M, n=172 and n=83 for controls and cases respectively
† Covariates being adjusted for were sex, age, systolic blood pressure, smoking, body mass index, triglycerides, high-density lipoprotein (HDL) cholesterol, non-HDL cholesterol, C-reactive protein (CRP), use of anti-hypertensive and lipid-lowering medication, and presence of chronic disease

For peer review only

Table 3. Baseline characteristics of 125 patients with acute MI in the APACHE cohort

Characteristics	Total (n=125)	STEMI (n=73)	NSTEMI (n=52)	*p*
Age, years	66.0 (12)	65.9 (13)	66.1 (11)	0.86
Women, n (%)	37 (30)	25 (34)	12 (23)	0.18
Smokers, n (%)	34 (27)	25 (34)	9 (17)	0.037
BMI, kg/m^2 [a]	26.3 (4.4)	26.2 (4.2)	26.3 (4.1)	0.59
Systolic blood pressure, mmHg	148 (28)	141 (26)	159 (28)	0.001
Diastolic blood pressure, mmHg	83 (17)	81 (15)	86 (20)	0.14
Lipid-lowering medication, n (%) [b]	31 (25)	13 (18)	18 (35)	0.033
Diabetes, n (%)	16 (13)	7 (9.5)	9 (17)	0.18
LV-function, normal/mildly reduced, n (%)	109 (87)	60 (82)	49 (94)	<0.001
hsTnT (at 6-8 h), ng/mL [a]	9.12 (91)	7.90 (130)	17.5 (71)	0.68
HDL-cholesterol, mmol/L [a]	1.10 (0.37)	1.10 (0.39)	1.10 (0.45)	0.59
LDL-cholesterol, mmol/L	2.84 (0.90)	2.94 (0.89)	2.67 (0.89)	0.12
Non-HDL cholesterol, mmol/L [a]	3.49 (1.3)	3.50 (1.3)	3.40 (1.5)	0.51
Triglycerides, mmol/L [a]	1.45 (0.87)	1.40 (0.75)	1.50 (1.1)	0.50
CRP, mg/L [a]	3.66 (6.0)	4.44 (5.3)	3.16 (7.0)	0.34
IL-6, pg/mL [a]	3.58 (5.0)	4.36 (5.8)	2.64 (3.0)	0.005
MMP-9, ng/mL [a]	263 (210)	253 (170)	310 (230)	0.62
COL1α1, pg/mL [a]	3590 (3200)	3670 (3900)	3580 (2400)	0.31

BMI = body mass index; hsTnT = high sensitivity Troponin T; HDL = high density lipoprotein; CRP = C-reactive protein; IL-6 = interleukin-6; MMP-9 = matrix metalloproteinase-9; COL1α1 = collagen type I α1 chain.

Statistics expressed as mean (SD or median (IQR)[a] if data do not possess distribution of normality. P-values from comparing STEMI and NSTEMI patients in terms of characteristics. Laboratory variables represent levels at admission (if not otherwise indicated).

[b] Defined as statin use at arrival to cardiology department

For peer review only

For peer review only - http://bmjopen.bmj.com/site/about/guidelines.xhtml

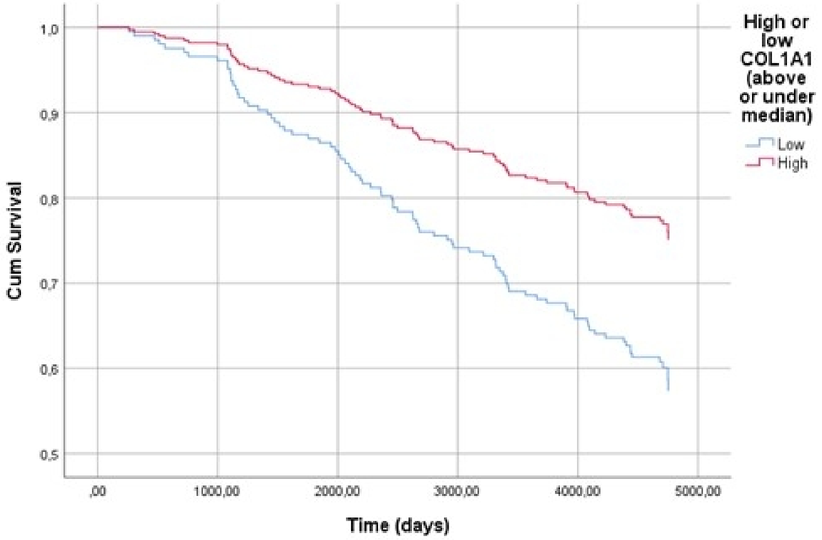

Figure 1a. Kaplan Meier survival curve showing cumulative survival in terms of primary outcome (MI or invasive coronary intervention) between participants with high or low COL1α1 values (above or under median) in the LSH cohort (COL1α1 = collagen type I α1 chain; Cum = cumulative)

157x92mm (96 x 96 DPI)

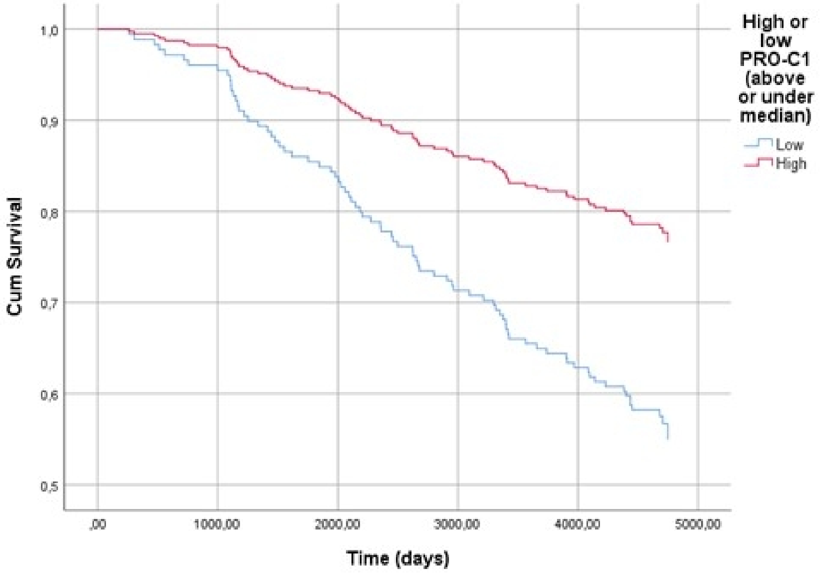

Figure 1b. Kaplan Meier survival curve showing cumulative survival in terms of primary outcome (MI or invasive coronary intervention) between participants with high or low PRO-C1 values (above or under median) in the LSH cohort (COL1α1 = collagen type I α1 chain; Cum = cumulative)

154x91mm (96 x 96 DPI)

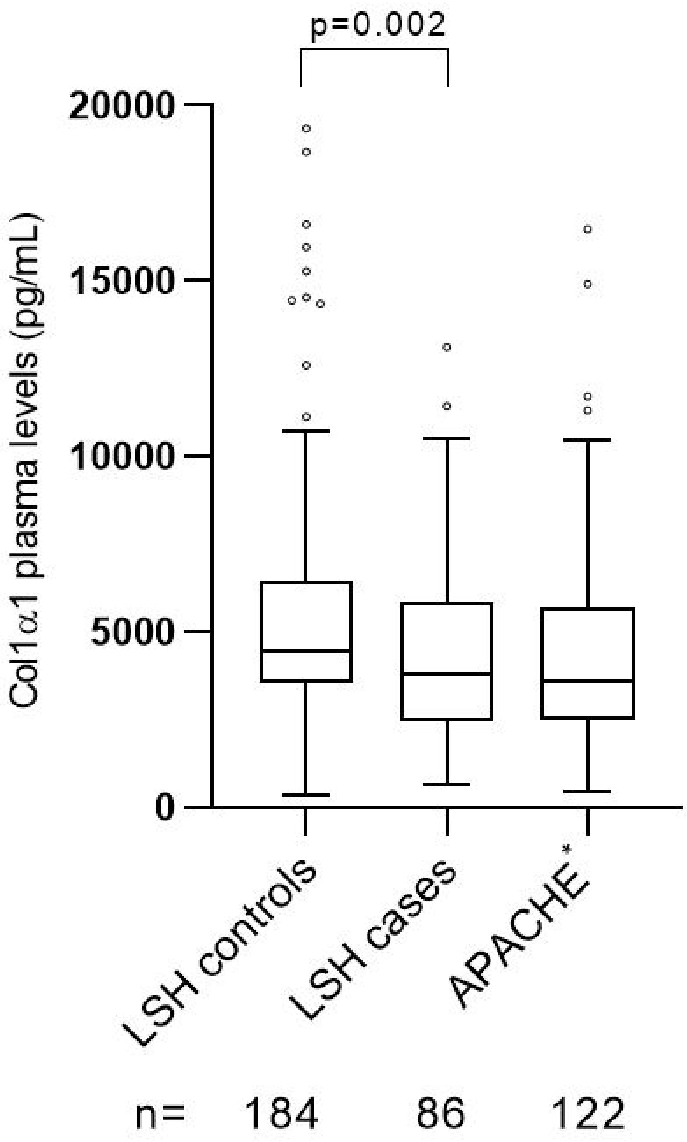

Figure 2. Box plots showing COL1α1 plasma levels (pg/mL) measured by the Luminex® Assay among participants in LSH and APACHE. P-value based on Mann-Whitney U test (APACHE = Assessing Platelet Activity in Coronary Heart Disease; COL1α1 = collagen type I α1 chain; LSH = Lifeconditions, Stress and Health study)
*At admission

73x120mm (144 x 144 DPI)

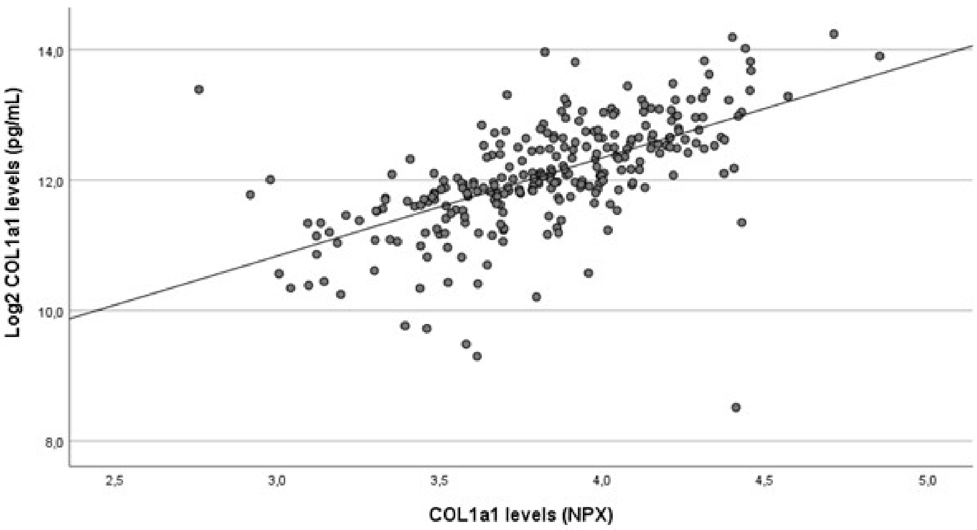

Supplementary figure 1. Scatter plot showing COL1α1 plasma levels in LSH participants based on Luminex-analysis (y-axis, log2) versus PEA-analysis (x-axis) along with linear regression line (COL1α1 = collagen type I α1 chain; Log2 = logarithmized with the base 2; NPX = normalized protein expression)

160x83mm (96 x 96 DPI)

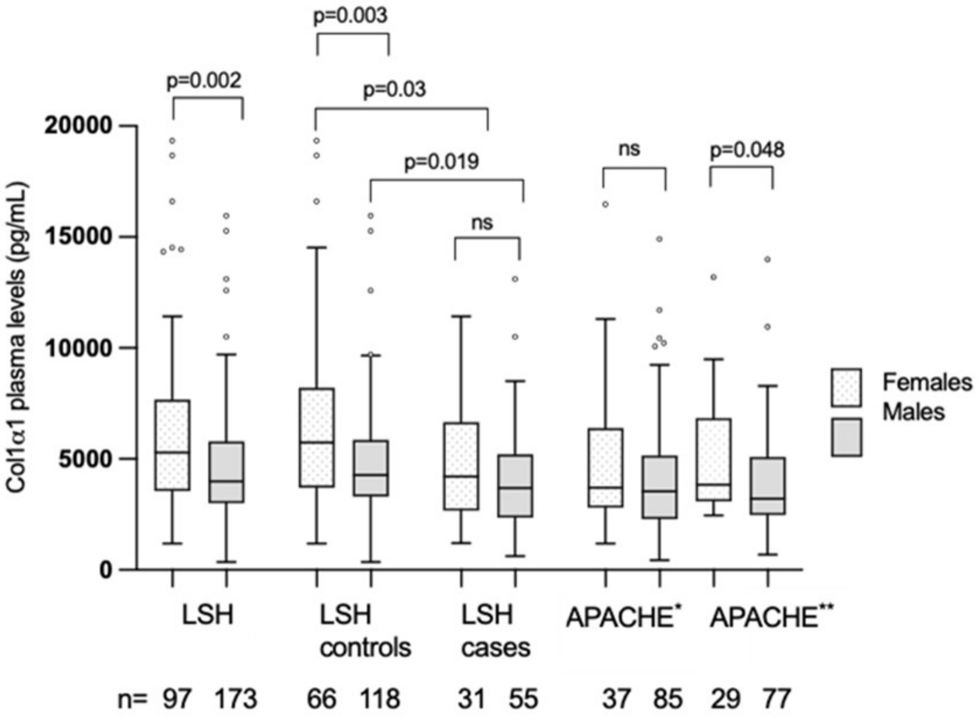

Supplementary figure 2. Box plots showing COL1α1 plasma levels (pg/mL) measured by the Luminex® Assay in LSH and APACHE cohorts split on sex. P-values based upon Mann-Whitney U tests (APACHE = Assessing Platelet Activity in Coronary Heart Disease; COL1α1 = collagen type I α1 chain; LSH = Lifeconditions, Stress and Health study)
*At admission
** 6 months after admission

145x107mm (150 x 150 DPI)

Supplementary table 1. Correlations between COL1α1, PRO-C1, C1M, demographic, clinical and laboratory variables in the LSH cohort expressed as correlation coefficients (*r*) and *p*-values

Variable	COL1α1		PRO-C1		C1M	
	r	*p*	*r*	*p*	*r*	*p*
Age	-0.01	*ns*	-0.08	*ns*	0.13	0.035
Sex[†]	0.19	0.002	0.21	0.001	0.13	0.045
BMI	-0.03	*ns*	-0.16	0.015	0.15	0.015
Diabetes[†]	-0.15	0.014	-0.09	*ns*	0.13	0.04
Angina pectoris[†]	-0.18	0.003	-0.17	0.007	0.08	*ns*
HDL cholesterol	0.13	0.029	0.19	0.003	-0.02	*ns*
Non-HDL cholesterol	0.07	*ns*	-0.04	*ns*	0.13	0.041
Triglycerides	-0.08	*ns*	-0.17	0.007	0.14	0.033
CRP	0.06	*ns*	0.01	*ns*	0.30	<0.001
IL-6	-0.05	*ns*	-0.16	0.017	0.21	0.001
MMP-9	-0.10	*ns*	-0.20	0.002	0.07	*ns*
Pro-C1	0.73	<0.001	-	-	-0.03	*ns*
C1M	<0.01	*ns*	-0.04	*ns*	-	-

COL1α1 = collagen type I α1 chain; BMI = body mass index; HDL = high density lipoprotein; CRP = C-reactive protein; IL-6 = interleukin-6; MMP-9 = matrix metalloproteinase-9; *ns* = non-significant
Correlations calculated with Spearman analysis. $p < 0.05$ considered statistically significant
[†] Male coded as 1 and female as 2, diabetes coded as 2 and not having diabetes coded as 1, angina pectoris coded as 2 and not having angina pectoris coded as 1

For peer review only

BMJ Open

Plasma type I collagen α1 chain in relation to coronary artery disease: findings from a prospective population-based cohort and an acute myocardial infarction prospective cohort in Sweden

Journal:	*BMJ Open*
Manuscript ID	bmjopen-2023-073561.R1
Article Type:	Original research
Date Submitted by the Author:	04-Aug-2023
Complete List of Authors:	Hammaréus, Filip; Linkoping University Department of Health Medicine and Caring Sciences<br>Nilsson, Lennart; Linkoping University Department of Health Medicine and Caring Sciences<br>Ong, Kwok-Leung; NHMRC Clinical Trials Centre<br>Kristenson, Margareta; Linkoping University Department of Health Medicine and Caring Sciences<br>Festin, Karin; Linkoping University Department of Health Medicine and Caring Sciences<br>Lundberg, Anna; Linkoping University Department of Health Medicine and Caring Sciences<br>Chung, Rosanna; Linkoping University Department of Health Medicine and Caring Sciences<br>Swahn, Eva; Linköping University, Department of Health, Medicine and Caring Science<br>Alfredsson, Joakim; Linkoping University Department of Health Medicine and Caring Sciences<br>Holm Nielsen, Signe; Technical University of Denmark Department of Biotechnology and Biomedicine; Nordic Bioscience<br>Jonasson, Lena; Linkoping University Department of Health Medicine and Caring Sciences
**Primary Subject Heading**:	Cardiovascular medicine
Secondary Subject Heading:	Cardiovascular medicine
Keywords:	Coronary heart disease < CARDIOLOGY, Vascular medicine < INTERNAL MEDICINE, Ischaemic heart disease < CARDIOLOGY

SCHOLARONE™
Manuscripts

I, the Submitting Author has the right to grant and does grant on behalf of all authors of the Work (as defined in the below author licence), an exclusive licence and/or a non-exclusive licence for contributions from authors who are: i) UK Crown employees; ii) where BMJ has agreed a CC-BY licence shall apply, and/or iii) in accordance with the terms applicable for US Federal Government officers or employees acting as part of their official duties; on a worldwide, perpetual, irrevocable, royalty-free basis to BMJ Publishing Group Ltd ("BMJ") its licensees and where the relevant Journal is co-owned by BMJ to the co-owners of the Journal, to publish the Work in this journal and any other BMJ products and to exploit all rights, as set out in our licence.

The Submitting Author accepts and understands that any supply made under these terms is made by BMJ to the Submitting Author unless you are acting as an employee on behalf of your employer or a postgraduate student of an affiliated institution which is paying any applicable article publishing charge ("APC") for Open Access articles. Where the Submitting Author wishes to make the Work available on an Open Access basis (and intends to pay the relevant APC), the terms of reuse of such Open Access shall be governed by a Creative Commons licence – details of these licences and which Creative Commons licence will apply to this Work are set out in our licence referred to above.

Other than as permitted in any relevant BMJ Author's Self Archiving Policies, I confirm this Work has not been accepted for publication elsewhere, is not being considered for publication elsewhere and does not duplicate material already published. I confirm all authors consent to publication of this Work and authorise the granting of this licence.

For peer review only

Plasma type I collagen α1 chain in relation to coronary artery disease: findings from a prospective population-based cohort and an acute myocardial infarction prospective cohort in Sweden

Filip Hammaréus[a], Lennart Nilsson[a], Kwok Leung Ong[b], Margareta Kristenson[c], Karin Festin[c], Anna K Lundberg[d], Rosanna WS Chung[d], Eva Swahn[e], Joakim Alfredsson[e], Signe Holm Nielsen[fg†], Lena Jonasson[e†]

[a] Department of Medicine in Jönköping and department of Health, Medicine and Caring Sciences, Unit of Cardiovascular Medicine, Linköping University, Linköping, Sweden
[b] NHMRC Clinical Trials Centre, Faculty of Medicine and Health, University of Sydney, Sydney, Australia
[c] Department of Health, Medicine and Caring Sciences, Division of Society and Health, Linköping University, Linköping, Sweden
[d] Department of Health, Medicine and Caring Sciences, Unit of Cardiovascular Medicine, Linköping University, Linköping, Sweden
[e] Department of Cardiology in Linköping, and Department of Health, Medicine and Caring Sciences, Unit of Cardiovascular Medicine, Linköping University, Linköping, Sweden
[f] Biomedicine and Biotechnology, Technical University of Denmark, Lyngby, Denmark
[g] ImmunoScience, Nordic Bioscience, Herlev, Denmark
† Shared authorship

Corresponding author:
Lennart Nilsson
+46-73-270 38 44
Lennart.nilsson@liu.se
Länssjukhuset Ryhov, 551 85, Jönköping, Sweden

Word count main text: 3994.

For peer review only

Abstract

Objectives: to investigate the association between type I collagen α1 chain (COL1α1) levels and coronary artery disease (CAD) by using absolute quantification in plasma. Also, to investigate the correlates of COL1α1 to clinical characteristics and circulating markers of collagen metabolism.

Design: Life conditions, Stress and Health (LSH) study: prospective cohort study, here with a nested case-control design.
Assessing Platelet Activity in Coronary Heart Disease (APACHE) study: prospective cohort study.

Setting: LSH: primary care setting, southeast Sweden.
APACHE: cardiology department, university hospital, southeast Sweden.

Participants: LSH: 1 007 randomly recruited individuals aged 45-69 (50% female), exclusion criteria was serious disease. After 13 years follow-up, 86 cases with primary end-point were identified and sex-/age-matched to 184 controls.
APACHE: 125 myocardial infarction (MI) patients, 73 with ST-elevation MI and 52 with non-ST-elevation MI. Exclusion criteria: Intervention study participation, warfarin treatment and short life expectancy.

Primary and secondary outcome measures: Primary outcome was the association between baseline COL1α1 and first-time major event of CAD, defined as fatal/non-fatal MI or coronary revascularization after 13 years. Secondary outcomes were the association between the collagen biomarkers PRO-C1/C1M and CAD; temporal change of COL1α1 after acute MI up to 6 months and lastly, correlates between COL1α1 and patient characteristics along with circulating markers of collagen metabolism.

Results: COL1α1 levels were associated with CAD, both unadjusted (HR=0.69, 95% CI=0.56-0.87) and adjusted (HR=0.55, 95% CI=0.41-0.75). PRO-C1 was associated with CAD, unadjusted (HR=0.62, 95% CI=0.47-0.82) and adjusted (HR=0.61, 95% CI=0.43-0.86), while C1M was not. In MI patients, COL1α1 remained unchanged up to 6 months. COL1α1 was correlated to PRO-C1, but not to C1M.

Conclusions: Plasma COL1α1 was independently and inversely associated with CAD. Furthermore, COL1α1 appeared to reflect collagen synthesis but not degradation. Future studies are needed to confirm whether COL1α1 is a clinically useful biomarker of CAD.

Keywords
Atherosclerosis, Coronary artery disease, Extracellular matrix, Collagen

For peer review only - http://bmjopen.bmj.com/site/about/guidelines.xhtml

Strengths and limitations of this study

- The study includes two well-characterized cohorts that complement each other in elucidating the role of type I collagen α1 chain (COL1α1) in coronary artery disease (CAD).
- Absolute quantification of COL1α1 in plasma was used to consolidate its novel relationship with incident CAD, as compared to a previous study using a relative quantification method.
- The relationship to other biomarkers reflecting collagen synthesis and degradation, along with markers of inflammation and myocardial injury was investigated to further clarify the role of COL1α1 in CAD.
- Although adjustments for the most common cardiovascular risk factors were performed in our models, there might be residual confounding factors that explain the observed associations.
- The study design and limited sample size (especially women subjects) makes it difficult to draw any definitive mechanistic conclusions from our results along with limiting the generalizability to other populations.

For peer review only

For peer review only - http://bmjopen.bmj.com/site/about/guidelines.xhtml

Introduction

Cardiovascular disease is the leading cause of death and source of disease burden worldwide. Most clinical manifestations, such as coronary artery disease (CAD), are caused by atherosclerosis (1). Atherosclerosis is a complex and long-lasting process in which multiple factors such as lipid profile, inflammation and extracellular matrix (ECM) remodeling contribute to the phenotype of the atherosclerotic plaque. Whether a plaque develops into a vulnerable one, with increased risk of atherothrombotic complications, largely depends on its phenotype (2). The ECM remodeling process in the arterial wall involves the synthesis and breakdown of ECM proteins such as collagens, proteoglycans and elastin (3). Excessive or uncontrolled ECM remodeling in CAD has been proposed by previous studies showing that ECM-degrading enzymes, such as matrix metalloproteinases (MMPs), are upregulated in rupture-prone plaques (4-6). Also, circulating levels of MMPs, particularly MMP-9, have been shown to predict the risk of CAD (7, 8).

Type I collagen is the most abundant protein in the human body and expressed in numerous tissues including skin, bone, tendons, and arteries. It consists of two identical polypeptide α1 chains (COL1α1) and one α2 chain resulting in a triple helix molecule. Pro-collagen is the precursor to mature collagen in tissues and has three major domains: an N-terminal pro-collagen domain, a central triple helical domain and a C-terminal pro-collagen domain (9). During the collagen synthesis, the N- and C-terminal pro-collagens are cleaved off from the central domain. These fragments, including the PRO-C1 biomarker, can reflect the systemic synthesis of type I collagen when measured in plasma (10). Breakdown fragments of the triple helical domain on the other hand, including the C1M biomarker, can reflect systemic degradation of type I collagen (11). In atherosclerotic plaques, type I collagen comprises 60% of the total protein content and is the primary component of the fibrous cap (12, 13). The type I collagen content is associated with a stable plaque phenotype. Reversibly, reduced synthesis and/or increased degradation of type I collagen may lead to thinning of the fibrous cap and thereby increase the risk of plaque rupture (5, 14, 15). In line with this, circulating markers of collagen type I degradation have been shown to predict incident CAD (16, 17). Whether biomarkers reflecting collagen type I synthesis can be used to predict cardiovascular risk has been studied to a lesser extent. Recently, we performed an exploratory study using relative quantification of 184 cardiovascular disease-related proteins to identify plasma biomarkers that predicted CAD over a 13-year follow-up period in a population-based Swedish cohort (18). Interestingly, COL1α1 was significantly and inversely associated with the risk of first-time major event of CAD. It also appeared to be the only biomarker which remained significant after multivariate adjustments for common cardiovascular risk factors. This was the first study showcasing the relevance of COL1α1 levels for future CAD risk. However, it is yet to be examined if COL1α1 levels are associated with collagen synthesis/degradation, inflammation and myocardial injury. Also, there are no studies examining COL1α1 changes in the circulation right after an acute CAD event.

Here, the overall aim was to investigate COL1α1 in plasma as a novel biomarker of CAD in a broader context, using two distinct cohorts. In a population-based cohort, we assessed if plasma COL1α1 could be used to determine future risk of CAD. We also assessed whether PRO-C1 and C1M, two biomarkers reflecting type I collagen synthesis and degradation, respectively, were associated with COL1α1 and CAD in the same cohort. In a clinical cohort, we measured plasma COL1α1 in patients with acute myocardial infarction (MI) from admission to 6 months after discharge. In both cohorts, we investigated the correlates of COL1α1, including a number of demographic, clinical and laboratory variables.

For peer review only - http://bmjopen.bmj.com/site/about/guidelines.xhtml

Methods

Study population I: The LSH cohort

The longitudinal Life conditions, Stress and Health (LSH) study has been described previously (8, 18). In summary, the LSH cohort consisted of 1007 individuals, aged 45-69 years and evenly distributed by sex, that were randomly recruited from 10 healthcare centers in the County of Östergötland in Southeast Sweden. The participation rate was 62.6%. Exclusion criteria were serious physical or psychiatric disease. One participant was excluded due to pancreatic cancer. Data were collected from the end of 2003 to the middle of 2004, using health questionnaires and a visit to the primary healthcare center for health status, including blood pressure measurements, body mass index (BMI) calculation and collection of fasting venous samples. Venous samples were analyzed immediately for routine laboratory analytes including blood lipids. In the questionnaires, participants reported smoking status, medical history and prescription drugs. A composite variable called "chronic disease" was created including chronic lung disease, cancer, and kidney disease. The measurements of C-reactive protein (CRP) (n=250), interleukin (IL)-6 (n=263) and MMP-9 (n=251) in plasma have been described previously (8). The primary outcome measured during a 13-year follow-up was onset of first-time major CAD event, defined as fatal/non-fatal MI or coronary revascularization (percutaneous coronary intervention or coronary artery bypass surgery). The outcome data was collected from the Swedish Cause of Death Registry and the Registry of Hospital Admissions managed by the Swedish National Board of Health and Welfare. Six participants did not approve of the linkage of their data to the National Board of Health and Welfare and was therefore excluded from the follow-up. For this study, 86 cases with a primary outcome were identified, 14 on fatal MI, 53 on non-fatal MI, 10 on PCI and 9 on CABG. Mean time to event was 7.0 years (range 0.7 to 13 years). For a nested case control design, 184 age- and sex-matched controls were identified. Two to three controls of the same sex and born in the same year were randomly selected through computer software to match the cases.

Study population II: The APACHE cohort

The study of Assessing Platelet Activity in Coronary Heart Disease (APACHE) has been described previously (19). The APACHE cohort consisted of 125 MI patients undergoing coronary angiography, whereof 73 with ST-elevation MI (STEMI) and 52 with non-ST-elevation MI (NSTEMI), defined according to the global definition of MI (20). The patients were recruited between January 2009 and August 2011 at the University Hospital Linköping, Sweden. Exclusion criteria were that the patient: already participated in an intervention study, were treated with warfarin before admission, had short life expectancy (less than 6 months) and were unwilling to participate. Information on previous medical history, use of medication and smoking status was collected in a case report form at the time of inclusion. Coronary angiography was performed on all STEMI patients immediately after admission to hospital and in 98 % of NSTEMI patients within 72 hours. Left ventricular function was assessed by echocardiography 2-3 days after admission. Patients were classified into having normal, mildly reduced, moderately reduced or severely reduced ventricular function according to established American/European guidelines (21). Blood samples were collected at 4 time points: at admission (n=122), 3 days (n=117), 7-9 days (n=110) and 6 months after admission (n=106). Laboratory tests such as lipid profile and CRP were analyzed according to clinical routine between day 2-4 after admission. High sensitivity troponin T (hsTnT) was assessed 6-8 hours after admission. MMP-9 and IL-6 were analyzed in plasma collected from admission (n=102) according to manufacturer´s instructions using commercially available ELISA kits (R&D Systems, Minneapolis MN, USA).

Quantification of COL1α1 in plasma

For peer review only - http://bmjopen.bmj.com/site/about/guidelines.xhtml

Absolute plasma levels of COL1α1 were quantified in both LSH cases (n=86) and controls (n=184), and in the APACHE cohort (n=125). All samples were sodium heparin plasma, except 15 samples in the LSH cohort that were EDTA-plasma (3 cases, 12 controls). Plasma was stored in -70°C from the time of collection until analysis in 2019. After thawing, the sample tubes were centrifuged at 16 000 x g for 4 minutes right before analysis. A Magnetic Luminex® Assay (R&D Systems Inc., Minneapolis, MN, USA, Catalog Number LXSAHM) was used to quantify COL1α1 according to manufacturer's instructions. The Magnetic Luminex® Assay steps and principles have been described in detail elsewhere (22). An automatic plate washer, HydroFlex™ (Tecan Trading AG, Switzerland), was used for washing steps. The samples were analyzed on a Flexmap 3D analyzer (Luminex corp., Hertogenbosch, Netherlands) platform using the Bioplex manager software (Biorad) to create a standard curve with a five-parameter logistic curve fit. On every assay plate, with 96 wells per plate, a duplicate of control plasma was analyzed to calculate intra- and inter-assay coefficients of variation (CV). In the LSH samples, the intra-assay CV ranged from 0.4% to 7.0% with an inter-assay CV of 14.6%. In the APACHE samples, intra-assay CV ranged from <0.1% to 4.4% and inter-assay CV amounted to 21.1%. Lower limit of quantification (LLOQ) ranged between 735-750 pg/mL while upper limit of quantification (ULOQ) ranged between 175 475-176 100 pg/mL. Three samples in LSH (2 controls, 1 case) were below the LLOQ and excluded from analysis. The type of anticoagulant did not seem to affect plasma levels of COL1α1, e.g., in LSH controls, the levels in sodium heparin plasma (n=172) did not differ significantly from those in EDTA-plasma (n=12), median 4410 pg/mL *vs.* 4490 pg/mL ($p = 0.21$).

Relative measurement of COL1α1 in the LSH cases (n=86) and controls (n=184) was performed using proximity extension assay which has been described previously (18). In short, 184 cardiovascular disease-related proteins, one of which was COL1α1, were measured in plasma. The PEA-technique utilizes DNA oligonucleotide labeled antibody probes to quantify protein concentrations through real-time polymerase chain reaction technology. The protein concentrations are presented as normalized protein expression (NPX) values. Higher and lower NPX values correspond to higher and lower protein concentrations, respectively, although not being absolute concentration values.

Quantification of PRO-C1 and C1M in plasma

Two neo-epitope specific biomarkers targeting type I collagen formation (PRO-C1) and type I collagen degradation (C1M) were measured in 255 heparin plasma samples (83 cases and 172 controls) in the LSH cohort using competitive ELISAs developed by Nordic Bioscience (Herlev, Denmark) (10, 11).The inter- and intra-assay coefficients of variation were <15% and <10% respectively for both assays. In the case of PRO-C1, 3 samples were below LLOQ while C1M had 21 samples below LLOQ along with 1 sample above ULOQ, we excluded these samples from further analysis.

Statistical analysis

Descriptive statistics were used to yield means, medians, standard deviations (SDs) and interquartile ranges (IQR). Medians (IQR) were presented instead of mean (SD) if the variable did not follow distribution of normality in any of the groups presented. Kolmogorov-Smirnov test was used for testing the distribution of normality for the variables. If not stated otherwise, missing values for variables were in single digits and were handled by exclusion from analysis. For comparison between two groups, independent samples t-test was used for normally distributed variables and Mann Whitney U-test for non-normally distributed variables. Correlation analyses were performed using Spearman´s analysis. A multiple linear regression model was used when testing for independent predictors of

For peer review only - http://bmjopen.bmj.com/site/about/guidelines.xhtml

biomarker levels. Variables significantly correlated to biomarker levels in Spearman's analysis were included as non-dependent variables in these models. In APACHE, Friedman analysis was used to explore if there was a significant difference in COL1α1 level across the four time points. To investigate the hazard ratio (HR) for COL1α1, pro-C1 and C1M in terms of primary outcome (see definition above) in the LSH population, a Cox proportional hazard regression analysis was performed, both with and without covariates. In accordance with our previous study (18), covariates being adjusted for were established cardiovascular risk factors: sex, age, systolic blood pressure, smoking, body mass index, triglycerides, high-density lipoprotein (HDL) cholesterol, non-HDL cholesterol, CRP, use of anti-hypertensive and lipid-lowering medication, and presence of chronic disease. When performing the Cox proportional hazard analysis, we used the $\log_2$ value of COL1α1. Pro-C1 and C1M Cox proportional hazard analyses were performed with $\log_2$ values as well. When assessing the difference in cumulative survival in terms of our primary outcome between groups the log rank test was performed to test for statistical significance along with plotting a Kaplan Meier curve for visualization. Two-tailed $p < 0.05$ was used as cut off for statistical significance. IBM SPSS, release 28 (IBM Corporation, Armonk, New York) was used for all statistical analyses.

Patient and Public Involvement statement

At the time of conducting this research, patients and/or the public were not involved in the design, conduct, reporting or dissemination of the research.

For peer review only

Results

Plasma levels of COL1α1 and their association with incident CAD in the LSH cohort

Demographic, clinical and laboratory characteristics of the LSH cases (n=86) and controls (n=184) are presented in **Table 1**. The mean age was 61 years and around 64 % were men. The cases smoked more, had higher blood pressures, higher levels of non-HDL cholesterol, triglycerides, CRP and MMP-9 and lower levels of HDL cholesterol compared with controls. As shown in **Table 2**, the absolute concentrations of COL1α1 measured by Luminex were significantly lower in LSH cases compared with controls. Furthermore, the absolute concentrations of COL1α1 exhibited a significant correlation with the PEA-based relative concentrations of COL1α1 ($r = 0.69$, $p < 0.001$, see **Supplementary figure 1)**. The PEA-based data were previously published by Ong *et al.* (18).

We next proceeded to assess whether absolute concentrations of COL1α1 were associated with incident CAD at 13-year follow-up (**Table 2**). When performing Cox proportional hazard analysis, COL1α1 levels showed significant association with incident CAD in the unadjusted model (HR = 0.69, 95% CI = 0.56-0.87, $p = 0.001$). In the adjusted model, COL1α1 levels remained significantly associated with incident CAD (HR = 0.55, 95% CI = 0.41-0.75, $p < 0.001$). Furthermore, when the LSH participants were separated into two groups based on their COL1α1 values, above or below the median level (≥ or < 4010 pg/mL), their cumulative survival in terms of CAD outcome differed significantly ($p = 0.002$, **Figure 1**).

Plasma levels of PRO-C1 and C1M and their associations with incident CAD in the LSH cohort

As further shown in **Table 2**, PRO-C1 levels were significantly lower in cases compared to controls in LSH, while C1M levels did not differ significantly between the groups. PRO-C1 levels showed a significant association with CAD outcome both in the unadjusted (HR = 0.62, 95% CI = 0.47 – 0.82, $p = 0.001$) and adjusted model (HR = 0.61, 95% CI = 0.43 – 0.86, $p = 0.005$). On the other hand, C1M levels did not show any significant association with CAD outcome. Cumulative survival differed significantly in terms of CAD outcome when the LSH cohort was split based on PRO-C1 levels into groups with above or below median PRO-CI levels (≥ or < 71.9 ng/mL) ($p < 0.001$, **Figure 1**).

Temporal changes of plasma COL1α1 in the APACHE cohort

Demographic, clinical and laboratory characteristics of the APACHE cohort, 73 patients with STEMI and 52 patients with NSTEMI, are shown in **Table 3**. The mean age was 66 years and around 70% were men. The COL1α1 levels did not change significantly from admission to 6 months in neither STEMI nor NSTEMI patients. In terms of different time points, COL1α1 median (IQR) value in the APACHE cohort was 3590 (3200), 3780 (3100), 3570 (3300) and 3330 (2800) pg/mL at arrival, after 3 days, after 7-9 days and after 6 months, respectively ($p = 0.92$). As shown in **Figure 2**, COL1α1 levels in the APACHE cohort (at admission) were comparable to COL1α1 levels in the LSH cases.

Correlates of COL1α1, PRO-C1 and C1M

Among baseline characteristics in the LSH cohort, COL1α1 showed correlations with female sex, HDL cholesterol, diabetes and angina pectoris (**Supplementary table 1)**. In a multiple regression model, only female sex remained significantly associated with COL1α1 levels (*beta* = 0.21, $p = 0.001$). As further shown in **Supplementary table 1**, PRO-C1 correlated with female sex, BMI, angina pectoris, triglycerides, HDL cholesterol, IL-6 and MMP-9. In a multiple regression model, only female sex remained significantly associated with PRO-C1 levels (*beta* = 0.23, $p = 0.001$). Further, C1M correlated to female sex, age, BMI, triglycerides, non-HDL cholesterol, CRP, diabetes and IL-6.

For peer review only - http://bmjopen.bmj.com/site/about/guidelines.xhtml

However, in a multiple regression model, only CRP (*beta* = 1.68, $p < 0.001$) and non-HDL cholesterol (*beta* = 1.80, $p = 0.032$) remained significantly related to C1M levels. When examining the intercorrelations between COL1α1, PRO-C1 and C1M in the LSH cohort, PRO-C1 and COL1α1 showed a strong correlation ($r = 0.73$, $p < 0.001$) while C1M did not correlate to either COL1α1 or PRO-C1.

In the APACHE cohort, female sex was the only variable that showed a significant correlation with COL1α1 concentrations in bivariate correlation analyses, though only at 6 months ($r = 0.19$, $p = 0.047$). COL1α1 did not showcase any significant correlations to IL-6, MMP-9 or CRP in LSH nor in APACHE. Neither was it correlated to hsTnT or to left ventricular dysfunction in APACHE.

As shown in **Supplementary Figure 2**, women had significantly higher COL1α1 levels than men in the LSH cohort. Also, female controls had significantly higher COL1α1 levels than female cases. Furthermore, PRO-C1 levels were higher in women than in men, 85.6 (60) *vs* 67.3 (32) ng/mL ($p = 0.001$) while C1M levels did not differ between the sexes (data not shown). In the APACHE cohort, the difference in COL1α1 levels at admission between men and women did not reach significance but was significantly different at 6 months, median (IQR) 3200 (2600) *vs.* 3840 (3700) pg/mL, respectively ($p = 0.048$).

For peer review only

Discussion

The present study showed that COL1α1 in plasma was independently and inversely associated with future risk of CAD. PRO-C1, a marker for type I collagen synthesis, showed a similar relationship to CAD outcome while C1M, a marker for type I collagen degradation, did not. Further investigation of COL1α1 in a MI population showed that levels remained stable up to 6 months after the acute event without any relationship to inflammation or myocardial injury.

In the population-based cohort, COL1α1 levels in plasma were significantly lower in those who developed CAD compared to those who did not. A cox proportional hazard analysis confirmed the association between lower COL1α1 levels and CAD outcome, even after multiple adjustment. The results confirm, and extend, the previous findings by Ong *et al.* (18). However, in that explorative study, the measurements of COL1α1 were based on relative quantification, and it was therefore important to validate the results by a quantifying assay. Prior to the study by Ong *et al.* (18), there were no studies available on COL1α1 in a CAD setting, except for one study reporting that a COL1α1 gene polymorphism occurred more frequently in MI survivors, as compared to age-matched controls (23).

Similar to COL1α1, PRO-C1, a biomarker of type I collagen synthesis, was inversely associated with CAD in both unadjusted and adjusted models while C1M, a biomarker for type I collagen breakdown, did not show any association. In line with this, COL1α1 showed a strong correlation with PRO-C1 indicating that both are markers of type I collagen synthesis. COL1α1 is the α1 chain partly making up the type I collagen molecule, whilst PRO-C1 is the internal epitope in the N-terminal pro-peptide of type I collagen (PINP). Hence, they can be regarded as products from two different stages of type I collagen synthesis (9, 10). To our knowledge, no previous studies have investigated PRO-C1 in the context of CAD. On the other hand, C1M has been associated with increased risk of cardiovascular events. One previous study reported that elevated C1M levels predicted MI in a prospective cohort of postmenopausal women (n=5 450, median age 71 years) with a median follow-up time of 14 years (17). Another study with a follow-up of 6 years reported that elevated C1M levels predicted cardiovascular events and mortality in patients who had undergone carotid endarterectomy (n = 473, median age 72 years, 64% men) (16). The discrepancy in results between these previous studies and the present study as regards C1M could be due to several reasons. First, the two previous studies measured C1M in serum while we utilized plasma, a distinction that can lead to different outcomes with examples from other biomarkers such as MMPs (24). Moreover, the study populations are not comparable in terms of size and composition. For example, the study by Holm Nielsen *et al.* (16) included patients who had undergone carotid endarterectomy yielding an older cohort with high atherosclerotic burden compared to our population-based cohort.

In the present study, we also investigated the temporal pattern of COL1α1 levels in MI patients by performing serial measurements from admission up to 6 months after the acute event. COL1α1 did not show any temporal changes over time in STEMI or NSTEMI patients, neither did it show any correlation with inflammatory markers or hsTnT in the early phase. Obviously, COL1α1 levels in plasma were not associated with acute atherothrombosis, inflammation or myocardial injury. Interestingly, COL1α1 values in patients with acute MI in the APACHE cohort were found to be comparable to COL1α1 values in individuals who subsequently developed incident CAD in LSH, as shown in **Figure 2**. However, this comparison should be made with caution since it is based on two distinct cohorts.

For peer review only - http://bmjopen.bmj.com/site/about/guidelines.xhtml

Women had significantly higher levels of COL1α1 and PRO-C1 compared to men in the LSH cohort. Female sex was also the only determinant that remained independently associated with COL1α1 and PRO-C1 in a multiple regression model including baseline variables. Collagen homeostasis has been shown to be affected by estrogen (25). PRO-C1 and other bone turnover markers are known to increase in women after menopause while there is little increase in men with age (26). This may partly explain why women in our population-based cohort had higher levels of COL1α1 and PRO-C1 than men. The sex difference in COL1α1 was also observed in the APACHE cohort, however these data should be interpreted with caution due to the limited number of female subjects in APACHE.

The major limitation of our study is the limited sample size, especially as regards women. Also, our cohorts reflected the Swedish population, which may limit the generalizability of results to other populations. Furthermore, in the LSH-cohort, case-control matching was performed based only on age and sex, which might result in differences between the groups that could affect the study outcome. To address this issue, other factors known to be of major importance for CAD risk were included in adjustment models. Still, it is possible that we have residual confounding factors contributing to the association between COL1α1 and risk of CAD. A recent study showed that COL1α1 was upregulated in left ventricular heart tissue from heart failure patients and that plasma levels were increased in those who were candidates for heart transplant (27). However, the MI cohort in our study included only 16 patients with moderate or severe left ventricular dysfunction, which does not permit us to draw conclusions about the relationship between COL1α1 and heart failure.

Based on our results, it can be speculated that plasma levels of COL1α1 and PRO-C1 reflect the ability to form type I collagen in arterial tissue, thereby influencing the development and fate of atherosclerotic plaques and risk of MI. However, bones and tendons are other sources of COL1α1 in the circulation. Also, the liver may leak ECM components to the blood stream under pathological conditions (28). Whether COL1α1 itself has a causal role in the development of CAD is unclear. Interestingly, a few previous clinical studies have investigated the effect of collagen supplement therapies on atherosclerosis-related factors. These studies, although small and with short intervention periods, have shown positive effects on LDL cholesterol/HDL cholesterol ratio, toxic advanced glycation end-products, cardio-ankle vascular index and brachial-ankle pulse wave velocity (29, 30).

To summarize, we confirm that absolute concentrations of COL1α1 in plasma are independently and inversely associated with future risk of CAD. COL1α1 in plasma appears to reflect collagen synthesis, but not collagen degradation, inflammation or myocardial injury. Larger studies are needed to confirm the clinical usefulness of COL1α1 as a marker of coronary atherosclerosis and/or predictor of incident CAD.

For peer review only - http://bmjopen.bmj.com/site/about/guidelines.xhtml

Acknowledgments

We would like to acknowledge Bettina Jung and Sofie Madsen for their technical support. The research was supported by a grant from the County Council of Östergötland, Sweden, aimed towards scientists early in their career; Academy of Health Care, County Council of Jönköping (Futurum); and The Danish Research Foundation and Innovation Fund Denmark.

Competing interests

SHN is full-time employees at Nordic Bioscience A/S. Nordic Bioscience is a privately-owned, small–medium size enterprise (SME) partly focused on the development of biomarkers including PRO-C1 and C1M. No fees, bonuses or other benefits was rewarded for the work described in the manuscript. SHN hold stocks in Nordic Bioscience A/S.

Author contributions

Filip Hammaréus wrote the manuscript; Filip Hammaréus, Anna Lundberg, Lena Jonasson, Margareta Kristenson, Kwok Leung Ong, Eva Swahn and Joakim Alfredsson participated in study design; Filip Hammaréus, Rosanna WS Chung and Anna Kristina Lundberg prepared plasma samples and performed the Luminex-assay on COL1α1; Signe Holm Nielsen measured C1M and PRO-C1 levels in the LSH cohort; Filip Hammaréus, Karin Festin, Lennart Nilsson and Joakim Alfredsson participated in data analysis; All authors participated in data interpretation and critical revision of the manuscript.

Data availability statement

Data are available on reasonable request.

Funding source

The LSH study was funded by the Swedish Research Council (2004−1881) and the Swedish Heart and Lung Foundation (2004053). The APACHE study received financial support from ALF Region Östergotland (LIO 131 471). The research was supported by the County Council of Östergötland, specifically with a grant aimed towards scientists early in their career (RÖ-910951); Academy of Health Care, County Council of Jönköping (N/A); and The Danish Research Foundation (N/A) and Innovation Fund Denmark (N/A). Further, we acknowledge the support from Linköping university. The funding sources had no role in study design, execution, analysis, interpretation of results or decision to submit.

Ethical considerations

As previously described (8, 19), the LSH and APACHE studies have been approved by The Regional Ethical Review Board in Linköping (Dnr 02–0324 and M45-08 respectively). All study participants have given written informed consent.

For peer review only

References

1. Herrington W, Lacey B, Sherliker P, Armitage J, Lewington S. Epidemiology of Atherosclerosis and the Potential to Reduce the Global Burden of Atherothrombotic Disease. Circ Res. 2016;118(4):535-46.
2. Bentzon JF, Otsuka F, Virmani R, Falk E. Mechanisms of plaque formation and rupture. Circ Res. 2014;114(12):1852-66.
3. Rekhter MD. Collagen synthesis in atherosclerosis: too much and not enough. Cardiovasc Res. 1999;41(2):376-84.
4. Galis ZS, Khatri JJ. Matrix metalloproteinases in vascular remodeling and atherogenesis: the good, the bad, and the ugly. Circ Res. 2002;90(3):251-62.
5. Galis ZS, Sukhova GK, Lark MW, Libby P. Increased expression of matrix metalloproteinases and matrix degrading activity in vulnerable regions of human atherosclerotic plaques. J Clin Invest. 1994;94(6):2493-503.
6. Gough PJ, Gomez IG, Wille PT, Raines EW. Macrophage expression of active MMP-9 induces acute plaque disruption in apoE-deficient mice. J Clin Invest. 2006;116(1):59-69.
7. Blankenberg S, Rupprecht HJ, Poirier O, Bickel C, Smieja M, Hafner G, et al. Plasma concentrations and genetic variation of matrix metalloproteinase 9 and prognosis of patients with cardiovascular disease. Circulation. 2003;107(12):1579-85.
8. Garvin P, Jonasson L, Nilsson L, Falk M, Kristenson M. Plasma Matrix Metalloproteinase-9 Levels Predict First-Time Coronary Heart Disease: An 8-Year Follow-Up of a Community-Based Middle Aged Population. PLoS One. 2015;10(9):e0138290.
9. Ricard-Blum S. The collagen family. Cold Spring Harb Perspect Biol. 2011;3(1):a004978.
10. Leeming DJ, Larsen DV, Zhang C, Hi Y, Veidal SS, Nielsen RH, et al. Enzyme-linked immunosorbent serum assays (ELISAs) for rat and human N-terminal pro-peptide of collagen type I (PINP)--assessment of corresponding epitopes. Clin Biochem. 2010;43(15):1249-56.
11. Leeming D, He Y, Veidal S, Nguyen Q, Larsen D, Koizumi M, et al. A novel marker for assessment of liver matrix remodeling: an enzyme-linked immunosorbent assay (ELISA) detecting a MMP generated type I collagen neo-epitope (C1M). Biomarkers. 2011;16(7):616-28.
12. Murata K, Motayama T, Kotake C. Collagen types in various layers of the human aorta and their changes with the atherosclerotic process. Atherosclerosis. 1986;60(3):251-62.
13. Shami A, Gonçalves I, Hultgårdh-Nilsson A. Collagen and related extracellular matrix proteins in atherosclerotic plaque development. Curr Opin Lipidol. 2014;25(5):394-9.
14. Edsfeldt A, Gonçalves I, Grufman H, Nitulescu M, Dunér P, Bengtsson E, et al. Impaired fibrous repair: a possible contributor to atherosclerotic plaque vulnerability in patients with type II diabetes. Arterioscler Thromb Vasc Biol. 2014;34(9):2143-50.
15. Wang J, Geng YJ, Guo B, Klima T, Lal BN, Willerson JT, et al. Near-infrared spectroscopic characterization of human advanced atherosclerotic plaques. J Am Coll Cardiol. 2002;39(8):1305-13.
16. Holm Nielsen S, Tengryd C, Edsfeldt A, Brix S, Genovese F, Bengtsson E, et al. A biomarker of collagen type I degradation is associated with cardiovascular events and mortality in patients with atherosclerosis. J Intern Med. 2019;285(1):118-23.
17. Bertelsen DM, Neergaard JS, Bager CL, Nielsen SH, Secher NH, Svendsen JH, et al. Matrix Metalloproteinase Mediated Type I Collagen Degradation is an Independent

For peer review only

Predictor of Increased Risk of Acute Myocardial Infarction in Postmenopausal Women. Sci Rep. 2018;8(1):5371.
18. Ong KL, Chung RWS, Hui N, Festin K, Lundberg AK, Rye KA, et al. Usefulness of Certain Protein Biomarkers for Prediction of Coronary Heart Disease. Am J Cardiol. 2020;125(4):542-8.
19. Alfredsson J, Lindahl TL, Gustafsson KM, Janzon M, Jonasson L, Logander E, et al. Large early variation of residual platelet reactivity in Acute Coronary Syndrome patients treated with clopidogrel: results from Assessing Platelet Activity in Coronary Heart Disease (APACHE). Thromb Res. 2015;136(2):335-40.
20. Thygesen K, Alpert JS, White HD, Joint ESCAAHAWHFTFftRoMI. Universal definition of myocardial infarction. J Am Coll Cardiol. 2007;50(22):2173-95.
21. Lang RM, Bierig M, Devereux RB, Flachskampf FA, Foster E, Pellikka PA, et al. Recommendations for chamber quantification. Eur J Echocardiogr. 2006;7(2):79-108.
22. Zhang Y, Li X, Di YP. Fast and Efficient Measurement of Clinical and Biological Samples Using Immunoassay-Based Multiplexing Systems. Methods Mol Biol. 2020;2102:129-47.
23. Speer G, Szenthe P, Kosa JP, Tabak AG, Folhoffer A, Fuszek P, et al. Myocardial infarction is associated with Spl binding site polymorphism of collagen type 1A1 gene. Acta Cardiol. 2006;61(3):321-5.
24. Mannello F. Serum or plasma samples? The "Cinderella" role of blood collection procedures: preanalytical methodological issues influence the release and activity of circulating matrix metalloproteinases and their tissue inhibitors, hampering diagnostic trueness and leading to misinterpretation. Arterioscler Thromb Vasc Biol. 2008;28(4):611-4.
25. McRobb LS, McGrath KCY, Tsatralis T, Liong EC, Tan JTM, Hughes G, et al. Estrogen Receptor Control of Atherosclerotic Calcification and Smooth Muscle Cell Osteogenic Differentiation. Arterioscler Thromb Vasc Biol. 2017;37(6):1127-37.
26. Eastell R, Pigott T, Gossiel F, Naylor KE, Walsh JS, Peel NFA. DIAGNOSIS OF ENDOCRINE DISEASE: Bone turnover markers: are they clinically useful? Eur J Endocrinol. 2018;178(1):R19-r31.
27. Hua X, Wang YY, Jia P, Xiong Q, Hu Y, Chang Y, et al. Multi-level transcriptome sequencing identifies COL1A1 as a candidate marker in human heart failure progression. BMC Med. 2020;18(1):2.
28. Nallagangula KS, Nagaraj SK, Venkataswamy L, Chandrappa M. Liver fibrosis: a compilation on the biomarkers status and their significance during disease progression. Future Sci OA. 2018;4(1):FSO250.
29. Tomosugi N, Yamamoto S, Takeuchi M, Yonekura H, Ishigaki Y, Numata N, et al. Effect of Collagen Tripeptide on Atherosclerosis in Healthy Humans. J Atheroscler Thromb. 2017;24(5):530-8.
30. Igase M, Kohara K, Okada Y, Ochi M, Igase K, Inoue N, et al. A double-blind, placebo-controlled, randomised clinical study of the effect of pork collagen peptide supplementation on atherosclerosis in healthy older individuals. Biosci Biotechnol Biochem. 2018;82(5):893-5.

For peer review only - http://bmjopen.bmj.com/site/about/guidelines.xhtml

Figure legends

Figure 1. Kaplan Meier survival curves showing cumulative survival in terms of primary outcome (MI or invasive coronary intervention) between participants with high versus low (above or under median) COL1α1 or PRO-C1 values in the LSH cohort (COL1α1 = collagen type I α1 chain; Cum = cumulative)

Figure 2. Box plots showing COL1α1 plasma levels (pg/mL) measured by the Luminex® Assay among participants in LSH and APACHE. *P*-value based on Mann-Whitney U test (APACHE = Assessing Platelet Activity in Coronary Heart Disease; COL1α1 = collagen type I α1 chain; LSH = Lifeconditions, Stress and Health study)

*At admission

Supplementary figure 1. Scatter plot showing COL1α1 plasma levels in LSH participants based on Luminex-analysis (y-axis, $\log_2$) versus PEA-analysis (x-axis) along with linear regression line (COL1α1 = collagen type I α1 chain; Log2 = logarithmized with the base 2; NPX = normalized protein expression)

Supplementary figure 2. Box plots showing COL1α1 plasma levels (pg/mL) measured by the Luminex® Assay in LSH and APACHE cohorts split on sex. *P*-values based upon Mann-Whitney U tests (APACHE = Assessing Platelet Activity in Coronary Heart Disease; COL1α1 = collagen type I α1 chain; LSH = Lifeconditions, Stress and Health study)

*At admission

** 6 months after admission

For peer review only - http://bmjopen.bmj.com/site/about/guidelines.xhtml

Tables

Table 1. Baseline characteristics of cases and controls in the LSH cohort

Characteristics	Controls (n=184)	Cases (n=86)	*p*
Age, years	60.6 (6.4)	60.6 (6.6)	0.84
Women, n (%)	66 (36)	31 (36)	>0.9
Smokers, n (%)	19 (11)	24 (30)	<0.001
BMI, kg/m^2	26.8 (3.6)	27.8 (3.8)	0.048
Systolic blood pressure, mmHg	135 (18)	146 (24)	<0.001
Antihypertensive medication, n (%)	32 (17)	30 (35)	0.002
Lipid-lowering medication, n (%)	9 (4.9)	11 (13)	0.027
Diabetes, n (%)	14 (7.6)	8 (9.3)	0.64
Angina pectoris, n (%)	4 (2.2)	12 (14)	<0.001
Serious chronic illness, n (%)	13 (7.1)	9 (11)	0.34
HDL-cholesterol, mmol/L [a]	1.50 (0.50)	1.40 (0.42)	0.001
LDL-cholesterol, mmol/L	3.54 (0.82)	3.64 (0.90)	0.39
Non-HDL cholesterol, mmol/L	4.11 (0.89)	4.36 (0.91)	0.036
Triglycerides, mmol/L[a]	1.10 (0.66)	1.40 (0.92)	<0.001
CRP, mg/L [a]	0.835 (1.7)	1.56 (2.4)	0.01
IL-6, pg/mL [a]	0.560 (2.25)	1.68 (2.5)	0.097
MMP-9, ng/mL [a]	30.7 (32)	39.5 (31)	0.036

BMI = body mass index; HDL = high density lipoprotein; LDL = low density lipoprotein; CRP = C-reactive protein; IL-6 = interleukin-6; MMP-9 = matrix metalloproteinase-9
Data expressed as mean (SD) or median (IQR)[a] if data do not possess distribution of normality. *P*-values from comparing controls and cases in terms of characteristics.

For peer review only

Table 2. Circulating levels of COL1α1, PRO-C1, C1M and their association with incident coronary artery disease at 13-year follow-up in the LSH cohort

Biomarker	Median (IQR) level				Unadjusted model		Adjusted model†	
	Control (n=184*)	Case (n=86*)	Difference	*p*	HR (95% CI)	*p*	HR (95% CI)	*p*
COL1α1 (pg/mL)	4430 (2900)	3810 (3400)	16%	0.002	0.69 (0.56-0.87)	0.001	0.55 (0.41-0.75)	<0.001
PRO-C1 (ng/mL)	77.6 (43)	59.7 (39)	30%	<0.001	0.62 (0.47-0.82)	0.001	0.61 (0.43-0.86)	0.005
C1M (ng/mL)	22.0 (16)	23.9 (19)	8.6%	*ns*	1.57 (0.568-4.34)	NS	0.92 (0.29-3.37)	*ns*

COL1α1 = collagen type I α1 chain; PRO-C1 = N-terminal pro-peptide of type I collagen; C1M = MMP-mediated degradation of type I collagen; HR = hazard ratio; *ns* = non-significant

Association expressed as hazard ratios per one unit increase in log2-tranformed unit from Cox proportional hazard analysis

*For pro-C1 and C1M, n=172 and n=83 for controls and cases respectively
† Covariates being adjusted for were sex, age, systolic blood pressure, smoking, body mass index, triglycerides, high-density lipoprotein (HDL) cholesterol, non-HDL cholesterol, C-reactive protein (CRP), use of anti-hypertensive and lipid-lowering medication, and presence of chronic disease

For peer review only

Table 3. Baseline characteristics of 125 patients with acute MI in the APACHE cohort

Characteristics	Total (n=125)	STEMI (n=73)	NSTEMI (n=52)	*p*
Age, years	66.0 (12)	65.9 (13)	66.1 (11)	0.86
Women, n (%)	37 (30)	25 (34)	12 (23)	0.18
Smokers, n (%)	34 (27)	25 (34)	9 (17)	0.037
BMI, kg/m^2 [a]	26.3 (4.4)	26.2 (4.2)	26.3 (4.1)	0.59
Systolic blood pressure, mmHg	148 (28)	141 (26)	159 (28)	0.001
Diastolic blood pressure, mmHg	83 (17)	81 (15)	86 (20)	0.14
Lipid-lowering medication, n (%) [b]	31 (25)	13 (18)	18 (35)	0.033
Diabetes, n (%)	16 (13)	7 (9.5)	9 (17)	0.18
LV-function, normal/mildly reduced, n (%)	109 (87)	60 (82)	49 (94)	<0.001
hsTnT (at 6-8 h), ng/mL [a]	9.12 (91)	7.90 (130)	17.5 (71)	0.68
HDL-cholesterol, mmol/L [a]	1.10 (0.37)	1.10 (0.39)	1.10 (0.45)	0.59
LDL-cholesterol, mmol/L	2.84 (0.90)	2.94 (0.89)	2.67 (0.89)	0.12
Non-HDL cholesterol, mmol/L [a]	3.49 (1.3)	3.50 (1.3)	3.40 (1.5)	0.51
Triglycerides, mmol/L [a]	1.45 (0.87)	1.40 (0.75)	1.50 (1.1)	0.50
CRP, mg/L [a]	3.66 (6.0)	4.44 (5.3)	3.16 (7.0)	0.34
IL-6, pg/mL [a]	3.58 (5.0)	4.36 (5.8)	2.64 (3.0)	0.005
MMP-9, ng/mL [a]	263 (210)	253 (170)	310 (230)	0.62
COL1α1, pg/mL [a]	3590 (3200)	3670 (3900)	3580 (2400)	0.31

BMI = body mass index; hsTnT = high sensitivity Troponin T; HDL = high density lipoprotein; CRP = C-reactive protein; IL-6 = interleukin-6; MMP-9 = matrix metalloproteinase-9; COL1α1 = collagen type I α1 chain.

Statistics expressed as mean (SD or median (IQR)[a] if data do not possess distribution of normality. P-values from comparing STEMI and NSTEMI patients in terms of characteristics. Laboratory variables represent levels at admission (if not otherwise indicated).

[b] Defined as statin use at arrival to cardiology department

For peer review only

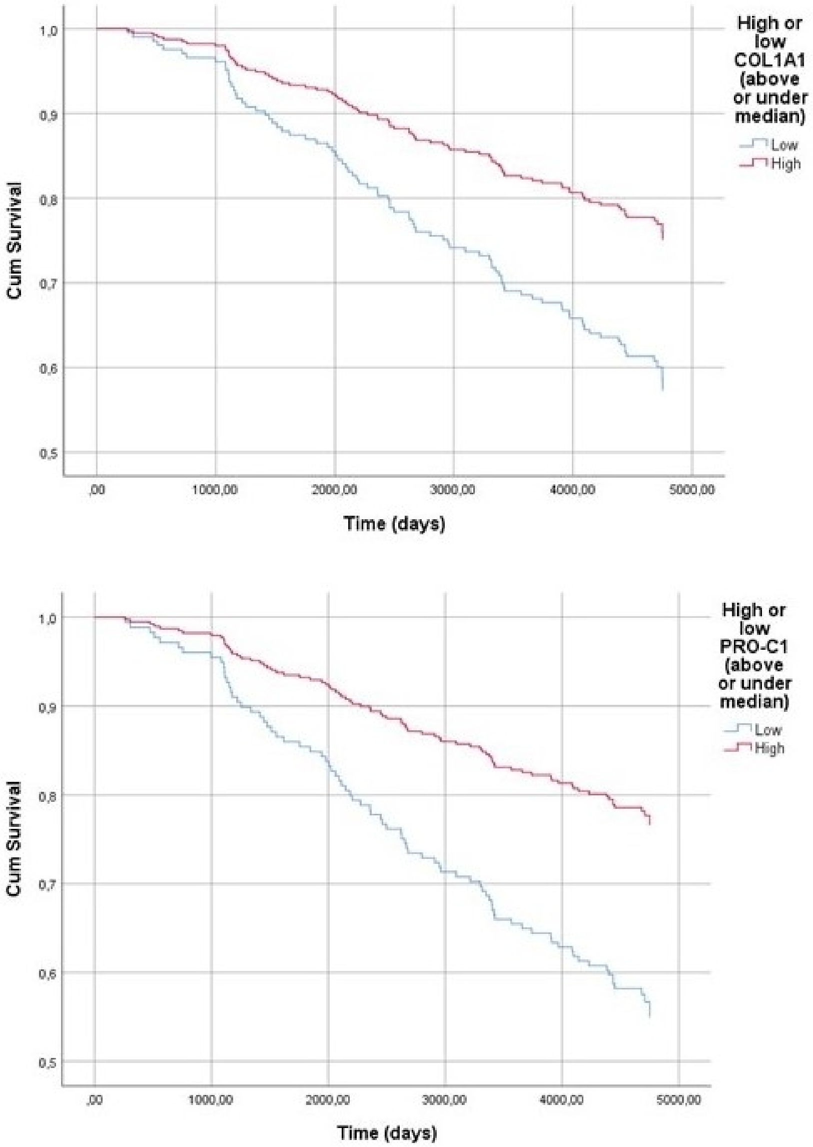

Kaplan Meier survival curves showing cumulative survival in terms of primary outcome (MI or invasive coronary intervention) between participants with high versus low (above or under median) COL1α1 or PRO-C1 values in the LSH cohort (COL1α1 = collagen type I α1 chain; Cum = cumulative)

137x189mm (96 x 96 DPI)

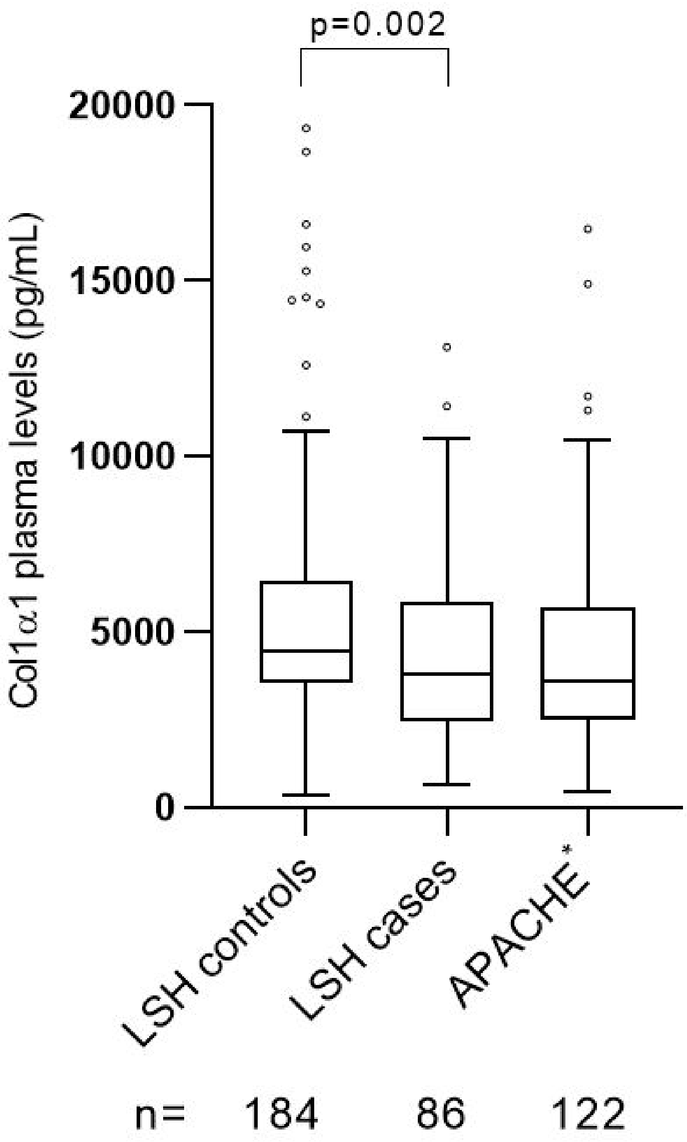

Figure 2. Box plots showing COL1α1 plasma levels (pg/mL) measured by the Luminex® Assay among participants in LSH and APACHE. P-value based on Mann-Whitney U test (APACHE = Assessing Platelet Activity in Coronary Heart Disease; COL1α1 = collagen type I α1 chain; LSH = Lifeconditions, Stress and Health study)
*At admission

73x120mm (144 x 144 DPI)

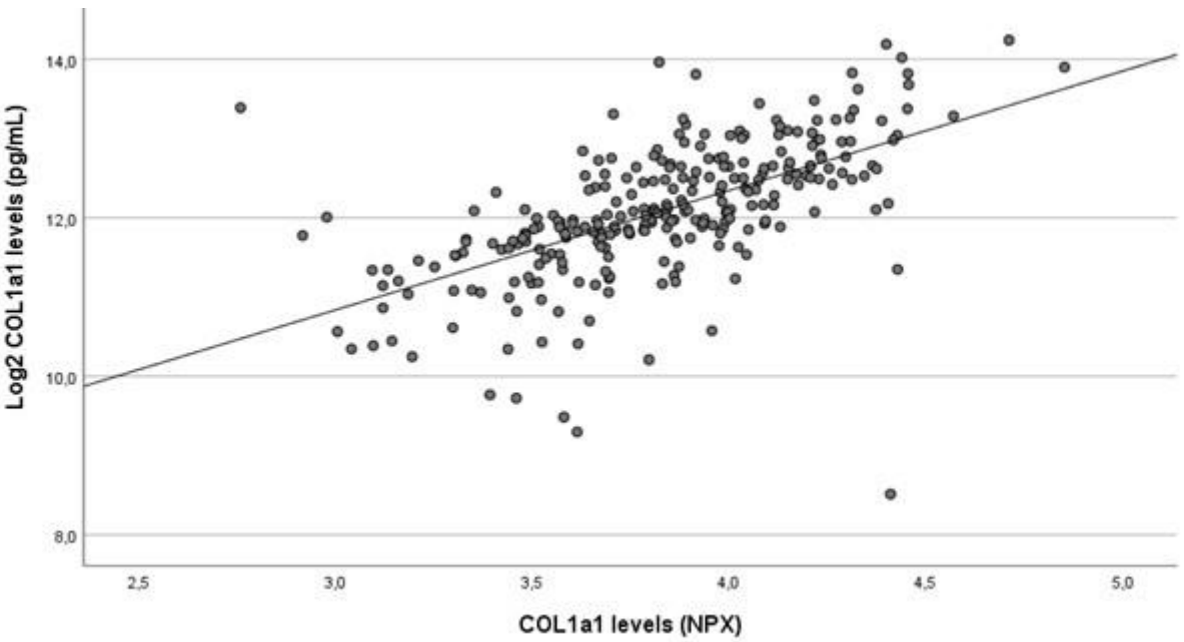

Supplementary figure 1. Scatter plot showing COL1α1 plasma levels in LSH participants based on Luminex-analysis (y-axis, log_2) versus PEA-analysis (x-axis) along with linear regression line (COL1α1 = collagen type I α1 chain; Log2 = logarithmized with the base 2; NPX = normalized protein expression)

peer review only

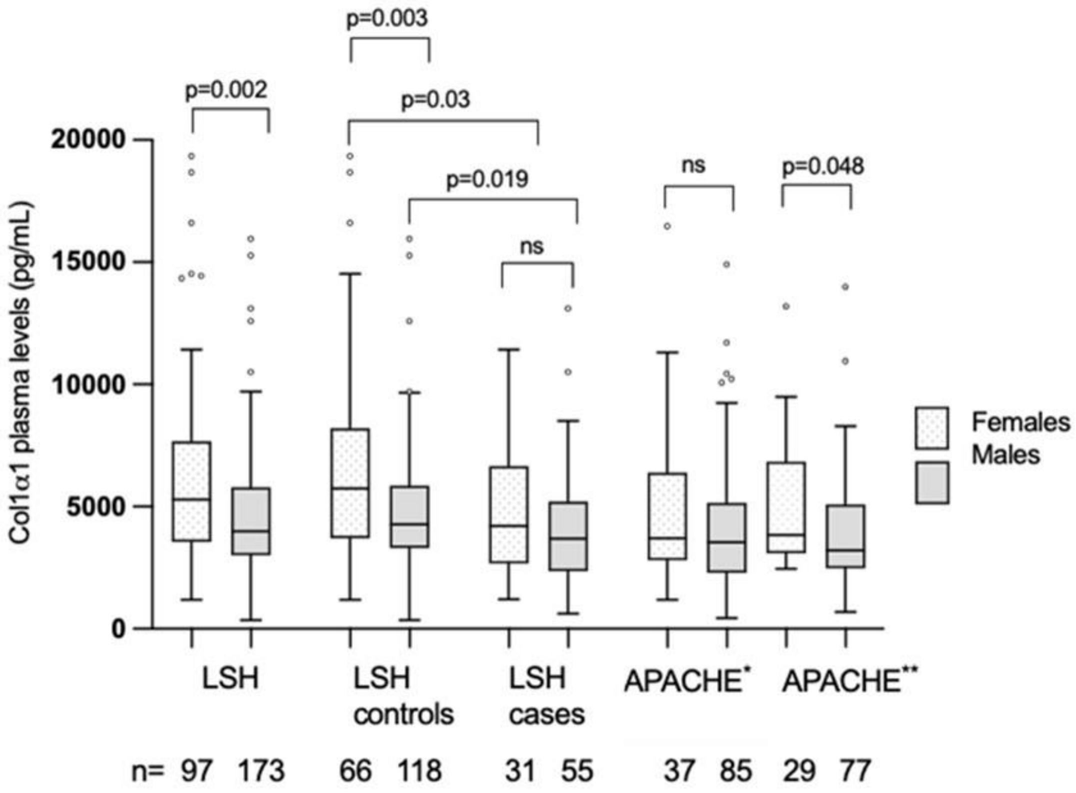

Supplementary figure 2. Box plots showing COL1α1 plasma levels (pg/mL) measured by the Luminex® Assay in LSH and APACHE cohorts split on sex. *P*-values based upon Mann-Whitney U tests (APACHE = Assessing Platelet Activity in Coronary Heart Disease; COL1α1 = collagen type I α1 chain; LSH = Lifeconditions, Stress and Health study)

*At admission

** 6 months after admission

Supplementary table 1. Correlations between COL1α1, PRO-C1, C1M, demographic, clinical and laboratory variables in the LSH cohort expressed as correlation coefficients (*r*) and *p*-values

Variable	COL1α1		PRO-C1		C1M	
	r	*p*	*r*	*p*	*r*	*p*
Age	-0.01	*ns*	-0.08	*ns*	0.13	0.035
Sex[†]	0.19	0.002	0.21	0.001	0.13	0.045
BMI	-0.03	*ns*	-0.16	0.015	0.15	0.015
Diabetes[†]	-0.15	0.014	-0.09	*ns*	0.13	0.04
Angina pectoris[†]	-0.18	0.003	-0.17	0.007	0.08	*ns*
HDL cholesterol	0.13	0.029	0.19	0.003	-0.02	*ns*
Non-HDL cholesterol	0.07	*ns*	-0.04	*ns*	0.13	0.041
Triglycerides	-0.08	*ns*	-0.17	0.007	0.14	0.033
CRP	0.06	*ns*	0.01	*ns*	0.30	<0.001
IL-6	-0.05	*ns*	-0.16	0.017	0.21	0.001
MMP-9	-0.10	*ns*	-0.20	0.002	0.07	*ns*
Pro-C1	0.73	<0.001	-	-	-0.03	*ns*
C1M	<0.01	*ns*	-0.04	*ns*	-	-

COL1α1 = collagen type I α1 chain; BMI = body mass index; HDL = high density lipoprotein; CRP = C-reactive protein; IL-6 = interleukin-6; MMP-9 = matrix metalloproteinase-9; *ns* = non-significant
Correlations calculated with Spearman analysis. $p < 0.05$ considered statistically significant
[†] Male coded as 1 and female as 2, diabetes coded as 2 and not having diabetes coded as 1, angina pectoris coded as 2 and not having angina pectoris coded as 1

For peer review only

STROBE Statement—checklist of items that should be included in reports of observational studies

All references to pages and lines are based on the manuscript version where tracked changes are remaining.

	Item No	Recommendation
**Title and abstract**	1	(*a*) Indicate the study's design with a commonly used term in the title or the abstract Page 1, line 1-3 (added prospective to enhance the description of the 2nd cohort) + page 2 line 53-55
		(*b*) Provide in the abstract an informative and balanced summary of what was done and what was found Page 2
**Introduction**		
Background/rationale	2	Explain the scientific background and rationale for the investigation being reported Page 4 line 108-143.
Objectives	3	State specific objectives, including any prespecified hypotheses Page 4-5 line 145-153
**Methods**		
Study design	4	Present key elements of study design early in the paper Page 6
Setting	5	Describe the setting, locations, and relevant dates, including periods of recruitment, exposure, follow-up, and data collection Page 5 + page 6 line 209-210
Participants	6	(*a*) *Cohort study*—Give the eligibility criteria, and the sources and methods of selection of participants. Describe methods of follow-up Page 6 line 160-163 + line 171-175 + line 186-191 + line 197-198<br>*Case-control study*—Give the eligibility criteria, and the sources and methods of case ascertainment and control selection. Give the rationale for the choice of cases and controls Page 6 line 160-163 + line 171-175 + line 177-182 + page 5 line 135-142<br>*Cross-sectional study*—Give the eligibility criteria, and the sources and methods of selection of participants Not applicable
		(*b*) *Cohort study*—For matched studies, give matching criteria and number of exposed and unexposed Not applicable<br>*Case-control study*—For matched studies, give matching criteria and the number of controls per case Page 6 line 179-181
Variables	7	Clearly define all outcomes, exposures, predictors, potential confounders, and effect modifiers. Give diagnostic criteria, if applicable Page 6 line 163-181 + line 191-203
Data sources/ measurement	8*	For each variable of interest, give sources of data and details of methods of assessment (measurement). Describe comparability of assessment methods if there is more than one group Page 7 line 205-245. Same measurement method was used for cases and controls.
Bias	9	Describe any efforts to address potential sources of bias Page 8 line 259-265 + page 7 line 223-225
Study size	10	Explain how the study size was arrived at Page 6 line 160-162 + line 177-181 + line 185-189. Two already existing cohorts.
Quantitative variables	11	Explain how quantitative variables were handled in the analyses. If applicable, describe which groupings were chosen and why Page 7 line 248-251 + page 8 line 265-266
Statistical methods	12	(*a*) Describe all statistical methods, including those used to control for confounding Page 7-8, line 247-270
		(*b*) Describe any methods used to examine subgroups and interactions Table 1 (page 18) + Table 3 (page 20) + page 8 line 254-257

For peer review only - http://bmjopen.bmj.com/site/about/guidelines.xhtml

	(*c*) Explain how missing data were addressed Page 6 line 175-177 + page 7 line 222-223 + line 243-245 + page 7-8 line 251-252
	(*d*) *Cohort study*—If applicable, explain how loss to follow-up was addressed. Not applicable, no cases/controls were lost to follow-up.<br>*Case-control study*—If applicable, explain how matching of cases and controls was addressed. Page 6 line 179-182<br>*Cross-sectional study*—If applicable, describe analytical methods taking account of sampling strategy Not applicable
	(*e*) Describe any sensitivity analyses Page 7 line 223-225

Continued on next page

For peer review only

**Results**		
Participants	13*	(a) Report numbers of individuals at each stage of study—eg numbers potentially eligible, examined for eligibility, confirmed eligible, included in the study, completing follow-up, and analysed Page 6 line 160-163 + line 175-181. Number of individuals eligible and excluded from APACHE not available.
		(b) Give reasons for non-participation at each stage Page 6 line 162-163 + line 175-177. Not available for APACHE.
		(c) Consider use of a flow diagram Not applicable given two cohorts which are described previously.
Descriptive data	14*	(a) Give characteristics of study participants (eg demographic, clinical, social) and information on exposures and potential confounders Page 9-10 results section + Table 1-3 (page 18-20)
		(b) Indicate number of participants with missing data for each variable of interest Page 6 line 169-171 + line 197-201 + Page 7 line 222-223 + line 243-245 + page 7-8 line 251-252
		(c) *Cohort study*—Summarise follow-up time (eg, average and total amount) Page 6 line 171-172 + 178-179
Outcome data	15*	*Cohort study*—Report numbers of outcome events or summary measures over time Page 6 line 177-179
		Case-control study—Report numbers in each exposure category, or summary measures of exposure Not applicable (nested case-control design)
		Cross-sectional study—Report numbers of outcome events or summary measures Not applicable
Main results	16	(*a*) Give unadjusted estimates and, if applicable, confounder-adjusted estimates and their precision (eg, 95% confidence interval). Make clear which confounders were adjusted for and why they were included Page 8 line 261-265 + page 9 line 294-298 + 303-311 + Table 2 (page 19)
		(*b*) Report category boundaries when continuous variables were categorized Page 9 line 298-301 + 308-311 + page 6 line 195-197
		(*c*) If relevant, consider translating estimates of relative risk into absolute risk for a meaningful time-period. Not applicable.
Other analyses	17	Report other analyses done—eg analyses of subgroups and interactions, and sensitivity analyses Page 9-10 results section, table 1 and 3 (page 18,20)
**Discussion**		
Key results	18	Summarise key results with reference to study objectives Page 11 line 350-357
Limitations	19	Discuss limitations of the study, taking into account sources of potential bias or imprecision. Discuss both direction and magnitude of any potential bias Page 12 line 414-425
Interpretation	20	Give a cautious overall interpretation of results considering objectives, limitations, multiplicity of analyses, results from similar studies, and other relevant evidence Page 12 line 427-436 + previous paragraphs in discussion
Generalisability	21	Discuss the generalisability (external validity) of the study results Page 12 line 414-416
**Other information**		
Funding	22	Give the source of funding and the role of the funders for the present study and, if applicable, for the original study on which the present article is based Page 1 line 28-35

*Give information separately for cases and controls in case-control studies and, if applicable, for exposed and unexposed groups in cohort and cross-sectional studies.

For peer review only

For peer review only - http://bmjopen.bmj.com/site/about/guidelines.xhtml

Note: An Explanation and Elaboration article discusses each checklist item and gives methodological background and published examples of transparent reporting. The STROBE checklist is best used in conjunction with this article (freely available on the Web sites of PLoS Medicine at http://www.plosmedicine.org/, Annals of Internal Medicine at http://www.annals.org/, and Epidemiology at http://www.epidem.com/). Information on the STROBE Initiative is available at www.strobe-statement.org.

For peer review only

For peer review only - http://bmjopen.bmj.com/site/about/guidelines.xhtml

BMJ Open

Plasma type I collagen α1 chain in relation to coronary artery disease: findings from a prospective population-based cohort and an acute myocardial infarction prospective cohort in Sweden

Journal:	*BMJ Open*
Manuscript ID	bmjopen-2023-073561.R2
Article Type:	Original research
Date Submitted by the Author:	21-Aug-2023

Complete List of Authors:	Hammaréus, Filip; Linkoping University Department of Health Medicine and Caring Sciences<br>Nilsson, Lennart; Linkoping University Department of Health Medicine and Caring Sciences<br>Ong, Kwok-Leung; NHMRC Clinical Trials Centre<br>Kristenson, Margareta; Linkoping University Department of Health Medicine and Caring Sciences<br>Festin, Karin; Linkoping University Department of Health Medicine and Caring Sciences<br>Lundberg, Anna; Linkoping University Department of Health Medicine and Caring Sciences<br>Chung, Rosanna; Linkoping University Department of Health Medicine and Caring Sciences<br>Swahn, Eva; Linköping University, Department of Health, Medicine and Caring Science<br>Alfredsson, Joakim; Linkoping University Department of Health Medicine and Caring Sciences<br>Holm Nielsen, Signe; Technical University of Denmark Department of Biotechnology and Biomedicine; Nordic Bioscience<br>Jonasson, Lena; Linkoping University Department of Health Medicine and Caring Sciences

**Primary Subject Heading**:	Cardiovascular medicine
Secondary Subject Heading:	Cardiovascular medicine
Keywords:	Coronary heart disease < CARDIOLOGY, Vascular medicine < INTERNAL MEDICINE, Ischaemic heart disease < CARDIOLOGY

SCHOLARONE™
Manuscripts

I, the Submitting Author has the right to grant and does grant on behalf of all authors of the Work (as defined in the below author licence), an exclusive licence and/or a non-exclusive licence for contributions from authors who are: i) UK Crown employees; ii) where BMJ has agreed a CC-BY licence shall apply, and/or iii) in accordance with the terms applicable for US Federal Government officers or employees acting as part of their official duties; on a worldwide, perpetual, irrevocable, royalty-free basis to BMJ Publishing Group Ltd ("BMJ") its licensees and where the relevant Journal is co-owned by BMJ to the co-owners of the Journal, to publish the Work in this journal and any other BMJ products and to exploit all rights, as set out in our licence.

The Submitting Author accepts and understands that any supply made under these terms is made by BMJ to the Submitting Author unless you are acting as an employee on behalf of your employer or a postgraduate student of an affiliated institution which is paying any applicable article publishing charge ("APC") for Open Access articles. Where the Submitting Author wishes to make the Work available on an Open Access basis (and intends to pay the relevant APC), the terms of reuse of such Open Access shall be governed by a Creative Commons licence – details of these licences and which Creative Commons licence will apply to this Work are set out in our licence referred to above.

Other than as permitted in any relevant BMJ Author's Self Archiving Policies, I confirm this Work has not been accepted for publication elsewhere, is not being considered for publication elsewhere and does not duplicate material already published. I confirm all authors consent to publication of this Work and authorise the granting of this licence.

For peer review only

Plasma type I collagen α1 chain in relation to coronary artery disease: findings from a prospective population-based cohort and an acute myocardial infarction prospective cohort in Sweden

Filip Hammaréus[a], Lennart Nilsson[a], Kwok Leung Ong[b], Margareta Kristenson[c], Karin Festin[c], Anna K Lundberg[d], Rosanna WS Chung[d], Eva Swahn[e], Joakim Alfredsson[e], Signe Holm Nielsen[fg†], Lena Jonasson[e†]

[a] Department of Medicine in Jönköping and department of Health, Medicine and Caring Sciences, Unit of Cardiovascular Medicine, Linköping University, Linköping, Sweden
[b] NHMRC Clinical Trials Centre, Faculty of Medicine and Health, University of Sydney, Sydney, Australia
[c] Department of Health, Medicine and Caring Sciences, Division of Society and Health, Linköping University, Linköping, Sweden
[d] Department of Health, Medicine and Caring Sciences, Unit of Cardiovascular Medicine, Linköping University, Linköping, Sweden
[e] Department of Cardiology in Linköping, and Department of Health, Medicine and Caring Sciences, Unit of Cardiovascular Medicine, Linköping University, Linköping, Sweden
[f] Biomedicine and Biotechnology, Technical University of Denmark, Lyngby, Denmark
[g] ImmunoScience, Nordic Bioscience, Herlev, Denmark
† Shared authorship

Corresponding author:
Lennart Nilsson
+46-73-270 38 44
Lennart.nilsson@liu.se
Länssjukhuset Ryhov, 551 85, Jönköping, Sweden

Word count main text: 3994.

For peer review only

Abstract

Objectives: to investigate the association between type I collagen α1 chain (COL1α1) levels and coronary artery disease (CAD) by using absolute quantification in plasma. Also, to investigate the correlates of COL1α1 to clinical characteristics and circulating markers of collagen metabolism.

Design: Life conditions, Stress and Health (LSH) study: prospective cohort study, here with a nested case-control design.
Assessing Platelet Activity in Coronary Heart Disease (APACHE) study: prospective cohort study.

Setting: LSH: primary care setting, southeast Sweden.
APACHE: cardiology department, university hospital, southeast Sweden.

Participants: LSH: 1 007 randomly recruited individuals aged 45-69 (50% female), exclusion criteria was serious disease. After 13 years follow-up, 86 cases with primary end-point were identified and sex-/age-matched to 184 controls.
APACHE: 125 myocardial infarction (MI) patients, 73 with ST-elevation MI and 52 with non-ST-elevation MI. Exclusion criteria: Intervention study participation, warfarin treatment and short life expectancy.

Primary and secondary outcome measures: Primary outcome was the association between baseline COL1α1 and first-time major event of CAD, defined as fatal/non-fatal MI or coronary revascularization after 13 years. Secondary outcomes were the association between the collagen biomarkers PRO-C1/C1M and CAD; temporal change of COL1α1 after acute MI up to 6 months and lastly, correlates between COL1α1 and patient characteristics along with circulating markers of collagen metabolism.

Results: COL1α1 levels were associated with CAD, both unadjusted (HR=0.69, 95% CI=0.56-0.87) and adjusted (HR=0.55, 95% CI=0.41-0.75). PRO-C1 was associated with CAD, unadjusted (HR=0.62, 95% CI=0.47-0.82) and adjusted (HR=0.61, 95% CI=0.43-0.86), while C1M was not. In MI patients, COL1α1 remained unchanged up to 6 months. COL1α1 was correlated to PRO-C1, but not to C1M.

Conclusions: Plasma COL1α1 was independently and inversely associated with CAD. Furthermore, COL1α1 appeared to reflect collagen synthesis but not degradation. Future studies are needed to confirm whether COL1α1 is a clinically useful biomarker of CAD.

Keywords

Atherosclerosis, Coronary artery disease, Extracellular matrix, Collagen

For peer review only

Strengths and limitations of this study

- The study includes two well-characterized cohorts that complement each other in elucidating the role of type I collagen α1 chain (COL1α1) in coronary artery disease (CAD).
- Absolute quantification of COL1α1 in plasma was used to consolidate its novel relationship with incident CAD, as compared to a previous study using a relative quantification method.
- The relationship to other biomarkers reflecting collagen synthesis and degradation, along with markers of inflammation and myocardial injury was investigated to further clarify the role of COL1α1 in CAD.
- Although adjustments for the most common cardiovascular risk factors were performed in our models, there might be residual confounding factors that explain the observed associations.
- The study design and limited sample size (especially women subjects) makes it difficult to draw any definitive mechanistic conclusions from our results along with limiting the generalizability to other populations.

For peer review only

Introduction

Cardiovascular disease is the leading cause of death and source of disease burden worldwide. Most clinical manifestations, such as coronary artery disease (CAD), are caused by atherosclerosis (1). Atherosclerosis is a complex and long-lasting process in which multiple factors such as lipid profile, inflammation and extracellular matrix (ECM) remodeling contribute to the phenotype of the atherosclerotic plaque. Whether a plaque develops into a vulnerable one, with increased risk of atherothrombotic complications, largely depends on its phenotype (2). The ECM remodeling process in the arterial wall involves the synthesis and breakdown of ECM proteins such as collagens, proteoglycans and elastin (3). Excessive or uncontrolled ECM remodeling in CAD has been proposed by previous studies showing that ECM-degrading enzymes, such as matrix metalloproteinases (MMPs), are upregulated in rupture-prone plaques (4-6). Also, circulating levels of MMPs, particularly MMP-9, have been shown to predict the risk of CAD (7, 8).

Type I collagen is the most abundant protein in the human body and expressed in numerous tissues including skin, bone, tendons, and arteries. It consists of two identical polypeptide α1 chains (COL1α1) and one α2 chain resulting in a triple helix molecule. Pro-collagen is the precursor to mature collagen in tissues and has three major domains: an N-terminal pro-collagen domain, a central triple helical domain and a C-terminal pro-collagen domain (9). During the collagen synthesis, the N- and C-terminal pro-collagens are cleaved off from the central domain. These fragments, including the PRO-C1 biomarker, can reflect the systemic synthesis of type I collagen when measured in plasma (10). Breakdown fragments of the triple helical domain on the other hand, including the C1M biomarker, can reflect systemic degradation of type I collagen (11). In atherosclerotic plaques, type I collagen comprises 60% of the total protein content and is the primary component of the fibrous cap (12, 13). The type I collagen content is associated with a stable plaque phenotype. Reversibly, reduced synthesis and/or increased degradation of type I collagen may lead to thinning of the fibrous cap and thereby increase the risk of plaque rupture (5, 14, 15). In line with this, circulating markers of collagen type I degradation have been shown to predict incident CAD (16, 17). Whether biomarkers reflecting collagen type I synthesis can be used to predict cardiovascular risk has been studied to a lesser extent. Recently, we performed an exploratory study using relative quantification of 184 cardiovascular disease-related proteins to identify plasma biomarkers that predicted CAD over a 13-year follow-up period in a population-based Swedish cohort (18). Interestingly, COL1α1 was significantly and inversely associated with the risk of first-time major event of CAD. It also appeared to be the only biomarker which remained significant after multivariate adjustments for common cardiovascular risk factors. This was the first study showcasing the relevance of COL1α1 levels for future CAD risk. However, it is yet to be examined if COL1α1 levels are associated with collagen synthesis/degradation, inflammation and myocardial injury. Also, there are no studies examining COL1α1 changes in the circulation right after an acute CAD event.

Here, the overall aim was to investigate COL1α1 in plasma as a novel biomarker of CAD in a broader context, using two distinct cohorts. In a population-based cohort, we assessed if plasma COL1α1 could be used to determine future risk of CAD. We also assessed whether PRO-C1 and C1M, two biomarkers reflecting type I collagen synthesis and degradation, respectively, were associated with COL1α1 and CAD in the same cohort. In a clinical cohort, we measured plasma COL1α1 in patients with acute myocardial infarction (MI) from admission to 6 months after discharge. In both cohorts, we investigated the correlates of COL1α1, including a number of demographic, clinical and laboratory variables.

For peer review only - http://bmjopen.bmj.com/site/about/guidelines.xhtml

Methods

Study population I: The LSH cohort

The longitudinal Life conditions, Stress and Health (LSH) study has been described previously (8, 18). In summary, the LSH cohort consisted of 1007 individuals, aged 45-69 years and evenly distributed by sex, that were randomly recruited from 10 healthcare centers in the County of Östergötland in Southeast Sweden. The participation rate was 62.6%. Exclusion criteria were serious physical or psychiatric disease. One participant was excluded due to pancreatic cancer. Data were collected from the end of 2003 to the middle of 2004, using health questionnaires and a visit to the primary healthcare center for health status, including blood pressure measurements, body mass index (BMI) calculation and collection of fasting venous samples. Venous samples were analyzed immediately for routine laboratory analytes including blood lipids. In the questionnaires, participants reported smoking status, medical history and prescription drugs. A composite variable called "chronic disease" was created including chronic lung disease, cancer, and kidney disease. The measurements of C-reactive protein (CRP) (n=250), interleukin (IL)-6 (n=263) and MMP-9 (n=251) in plasma have been described previously (8). The primary outcome measured during a 13-year follow-up was onset of first-time major CAD event, defined as fatal/non-fatal MI or coronary revascularization (percutaneous coronary intervention or coronary artery bypass surgery). The outcome data was collected from the Swedish Cause of Death Registry and the Registry of Hospital Admissions managed by the Swedish National Board of Health and Welfare. Six participants did not approve of the linkage of their data to the National Board of Health and Welfare and was therefore excluded from the follow-up. For this study, 86 cases with a primary outcome were identified, 14 on fatal MI, 53 on non-fatal MI, 10 on PCI and 9 on CABG. Mean time to event was 7.0 years (range 0.7 to 13 years). For a nested case control design, 184 age- and sex-matched controls were identified. Two to three controls of the same sex and born in the same year were randomly selected through computer software to match the cases.

Study population II: The APACHE cohort

The study of Assessing Platelet Activity in Coronary Heart Disease (APACHE) has been described previously (19). The APACHE cohort consisted of 125 MI patients undergoing coronary angiography, whereof 73 with ST-elevation MI (STEMI) and 52 with non-ST-elevation MI (NSTEMI), defined according to the global definition of MI (20). The patients were recruited between January 2009 and August 2011 at the University Hospital Linköping, Sweden. Exclusion criteria were that the patient: already participated in an intervention study, were treated with warfarin before admission, had short life expectancy (less than 6 months) and were unwilling to participate. Information on previous medical history, use of medication and smoking status was collected in a case report form at the time of inclusion. Coronary angiography was performed on all STEMI patients immediately after admission to hospital and in 98 % of NSTEMI patients within 72 hours. Left ventricular function was assessed by echocardiography 2-3 days after admission. Patients were classified into having normal, mildly reduced, moderately reduced or severely reduced ventricular function according to established American/European guidelines (21). Blood samples were collected at 4 time points: at admission (n=122), 3 days (n=117), 7-9 days (n=110) and 6 months after admission (n=106). Laboratory tests such as lipid profile and CRP were analyzed according to clinical routine between day 2-4 after admission. High sensitivity troponin T (hsTnT) was assessed 6-8 hours after admission. MMP-9 and IL-6 were analyzed in plasma collected from admission (n=102) according to manufacturer´s instructions using commercially available ELISA kits (R&D Systems, Minneapolis MN, USA).

Quantification of COL1α1 in plasma

For peer review only - http://bmjopen.bmj.com/site/about/guidelines.xhtml

Absolute plasma levels of COL1α1 were quantified in both LSH cases (n=86) and controls (n=184), and in the APACHE cohort (n=125). All samples were sodium heparin plasma, except 15 samples in the LSH cohort that were EDTA-plasma (3 cases, 12 controls). Plasma was stored in -70°C from the time of collection until analysis in 2019. After thawing, the sample tubes were centrifuged at 16 000 x g for 4 minutes right before analysis. A Magnetic Luminex® Assay (R&D Systems Inc., Minneapolis, MN, USA, Catalog Number LXSAHM) was used to quantify COL1α1 according to manufacturer's instructions. The Magnetic Luminex® Assay steps and principles have been described in detail elsewhere (22). An automatic plate washer, HydroFlex™ (Tecan Trading AG, Switzerland), was used for washing steps. The samples were analyzed on a Flexmap 3D analyzer (Luminex corp., Hertogenbosch, Netherlands) platform using the Bioplex manager software (Biorad) to create a standard curve with a five-parameter logistic curve fit. On every assay plate, with 96 wells per plate, a duplicate of control plasma was analyzed to calculate intra- and inter-assay coefficients of variation (CV). In the LSH samples, the intra-assay CV ranged from 0.4% to 7.0% with an inter-assay CV of 14.6%. In the APACHE samples, intra-assay CV ranged from <0.1% to 4.4% and inter-assay CV amounted to 21.1%. Lower limit of quantification (LLOQ) ranged between 735-750 pg/mL while upper limit of quantification (ULOQ) ranged between 175 475-176 100 pg/mL. Three samples in LSH (2 controls, 1 case) were below the LLOQ and excluded from analysis. The type of anticoagulant did not seem to affect plasma levels of COL1α1, e.g., in LSH controls, the levels in sodium heparin plasma (n=172) did not differ significantly from those in EDTA-plasma (n=12), median 4410 pg/mL *vs.* 4490 pg/mL ($p = 0.21$).

Relative measurement of COL1α1 in the LSH cases (n=86) and controls (n=184) was performed using proximity extension assay which has been described previously (18). In short, 184 cardiovascular disease-related proteins, one of which was COL1α1, were measured in plasma. The PEA-technique utilizes DNA oligonucleotide labeled antibody probes to quantify protein concentrations through real-time polymerase chain reaction technology. The protein concentrations are presented as normalized protein expression (NPX) values. Higher and lower NPX values correspond to higher and lower protein concentrations, respectively, although not being absolute concentration values.

Quantification of PRO-C1 and C1M in plasma

Two neo-epitope specific biomarkers targeting type I collagen formation (PRO-C1) and type I collagen degradation (C1M) were measured in 255 heparin plasma samples (83 cases and 172 controls) in the LSH cohort using competitive ELISAs developed by Nordic Bioscience (Herlev, Denmark) (10, 11).The inter- and intra-assay coefficients of variation were <15% and <10% respectively for both assays. In the case of PRO-C1, 3 samples were below LLOQ while C1M had 21 samples below LLOQ along with 1 sample above ULOQ, we excluded these samples from further analysis.

Statistical analysis

Descriptive statistics were used to yield means, medians, standard deviations (SDs) and interquartile ranges (IQR). Medians (IQR) were presented instead of mean (SD) if the variable did not follow distribution of normality in any of the groups presented. Kolmogorov-Smirnov test was used for testing the distribution of normality for the variables. If not stated otherwise, missing values for variables were in single digits and were handled by exclusion from analysis. For comparison between two groups, independent samples t-test was used for normally distributed variables and Mann Whitney U-test for non-normally distributed variables. Correlation analyses were performed using Spearman´s analysis. A multiple linear regression model was used when testing for independent predictors of

For peer review only - http://bmjopen.bmj.com/site/about/guidelines.xhtml

biomarker levels. Variables significantly correlated to biomarker levels in Spearman's analysis were included as non-dependent variables in these models. In APACHE, Friedman analysis was used to explore if there was a significant difference in COL1α1 level across the four time points. To investigate the hazard ratio (HR) for COL1α1, pro-C1 and C1M in terms of primary outcome (see definition above) in the LSH population, a Cox proportional hazard regression analysis was performed, both with and without covariates. In accordance with our previous study (18), covariates being adjusted for were established cardiovascular risk factors: sex, age, systolic blood pressure, smoking, body mass index, triglycerides, high-density lipoprotein (HDL) cholesterol, non-HDL cholesterol, CRP, use of anti-hypertensive and lipid-lowering medication, and presence of chronic disease. When performing the Cox proportional hazard analysis, we used the $\log_2$ value of COL1α1. Pro-C1 and C1M Cox proportional hazard analyses were performed with $\log_2$ values as well. When assessing the difference in cumulative survival in terms of our primary outcome between groups the log rank test was performed to test for statistical significance along with plotting a Kaplan Meier curve for visualization. Two-tailed $p < 0.05$ was used as cut off for statistical significance. IBM SPSS, release 28 (IBM Corporation, Armonk, New York) was used for all statistical analyses.

Patient and Public Involvement statement

At the time of conducting this research, patients and/or the public were not involved in the design, conduct, reporting or dissemination of the research.

For peer review only - http://bmjopen.bmj.com/site/about/guidelines.xhtml

Results

Plasma levels of COL1α1 and their association with incident CAD in the LSH cohort

Demographic, clinical and laboratory characteristics of the LSH cases (n=86) and controls (n=184) are presented in **Table 1**. The mean age was 61 years and around 64 % were men. The cases smoked more, had higher blood pressures, higher levels of non-HDL cholesterol, triglycerides, CRP and MMP-9 and lower levels of HDL cholesterol compared with controls. As shown in **Table 2**, the absolute concentrations of COL1α1 measured by Luminex were significantly lower in LSH cases compared with controls. Furthermore, the absolute concentrations of COL1α1 exhibited a significant correlation with the PEA-based relative concentrations of COL1α1 ($r = 0.69$, $p < 0.001$, see **Supplementary figure 1)**. The PEA-based data were previously published by Ong *et al.* (18).

We next proceeded to assess whether absolute concentrations of COL1α1 were associated with incident CAD at 13-year follow-up (**Table 2**). When performing Cox proportional hazard analysis, COL1α1 levels showed significant association with incident CAD in the unadjusted model (HR = 0.69, 95% CI = 0.56-0.87, $p = 0.001$). In the adjusted model, COL1α1 levels remained significantly associated with incident CAD (HR = 0.55, 95% CI = 0.41-0.75, $p < 0.001$). Furthermore, when the LSH participants were separated into two groups based on their COL1α1 values, above or below the median level (≥ or < 4010 pg/mL), their cumulative survival in terms of CAD outcome differed significantly ($p = 0.002$, **Figure 1**).

Plasma levels of PRO-C1 and C1M and their associations with incident CAD in the LSH cohort

As further shown in **Table 2**, PRO-C1 levels were significantly lower in cases compared to controls in LSH, while C1M levels did not differ significantly between the groups. PRO-C1 levels showed a significant association with CAD outcome both in the unadjusted (HR = 0.62, 95% CI = 0.47 – 0.82, $p = 0.001$) and adjusted model (HR = 0.61, 95% CI = 0.43 – 0.86, $p = 0.005$). On the other hand, C1M levels did not show any significant association with CAD outcome. Cumulative survival differed significantly in terms of CAD outcome when the LSH cohort was split based on PRO-C1 levels into groups with above or below median PRO-CI levels (≥ or < 71.9 ng/mL) ($p < 0.001$, **Figure 1**).

Temporal changes of plasma COL1α1 in the APACHE cohort

Demographic, clinical and laboratory characteristics of the APACHE cohort, 73 patients with STEMI and 52 patients with NSTEMI, are shown in **Table 3**. The mean age was 66 years and around 70% were men. The COL1α1 levels did not change significantly from admission to 6 months in neither STEMI nor NSTEMI patients. In terms of different time points, COL1α1 median (IQR) value in the APACHE cohort was 3590 (3200), 3780 (3100), 3570 (3300) and 3330 (2800) pg/mL at arrival, after 3 days, after 7-9 days and after 6 months, respectively ($p = 0.92$). As shown in **Figure 2**, COL1α1 levels in the APACHE cohort (at admission) were comparable to COL1α1 levels in the LSH cases.

Correlates of COL1α1, PRO-C1 and C1M

Among baseline characteristics in the LSH cohort, COL1α1 showed correlations with female sex, HDL cholesterol, diabetes and angina pectoris (**Supplementary table 1)**. In a multiple regression model, only female sex remained significantly associated with COL1α1 levels (*beta* = 0.21, $p = 0.001$). As further shown in **Supplementary table 1**, PRO-C1 correlated with female sex, BMI, angina pectoris, triglycerides, HDL cholesterol, IL-6 and MMP-9. In a multiple regression model, only female sex remained significantly associated with PRO-C1 levels (*beta* = 0.23, $p = 0.001$). Further, C1M correlated to female sex, age, BMI, triglycerides, non-HDL cholesterol, CRP, diabetes and IL-6.

For peer review only - http://bmjopen.bmj.com/site/about/guidelines.xhtml

However, in a multiple regression model, only CRP (*beta* = 1.68, $p < 0.001$) and non-HDL cholesterol (*beta* = 1.80, $p = 0.032$) remained significantly related to C1M levels. When examining the intercorrelations between COL1α1, PRO-C1 and C1M in the LSH cohort, PRO-C1 and COL1α1 showed a strong correlation ($r = 0.73$, $p < 0.001$) while C1M did not correlate to either COL1α1 or PRO-C1.

In the APACHE cohort, female sex showed a significant but weak correlation with COL1α1 concentrations in bivariate correlation analyses at 6 months ($r = 0.19$, $p = 0.047$). COL1α1 did not showcase any significant correlations to IL-6, MMP-9 or CRP in LSH nor in APACHE. Neither was it correlated to hsTnT or to left ventricular dysfunction in APACHE.

As shown in **Supplementary Figure 2**, women had significantly higher COL1α1 levels than men in the LSH cohort. Also, female controls had significantly higher COL1α1 levels than female cases. Furthermore, PRO-C1 levels were higher in women than in men, 85.6 (60) *vs* 67.3 (32) ng/mL ($p = 0.001$) while C1M levels did not differ between the sexes (data not shown). In the APACHE cohort, the difference in COL1α1 levels at admission between men and women did not reach significance but was significantly different at 6 months, median (IQR) 3200 (2600) *vs.* 3840 (3700) pg/mL, respectively ($p = 0.048$).

For peer review only

For peer review only - http://bmjopen.bmj.com/site/about/guidelines.xhtml

Discussion

The present study showed that COL1α1 in plasma was independently and inversely associated with future risk of CAD. PRO-C1, a marker for type I collagen synthesis, showed a similar relationship to CAD outcome while C1M, a marker for type I collagen degradation, did not. Further investigation of COL1α1 in a MI population showed that levels remained stable up to 6 months after the acute event without any relationship to inflammation or myocardial injury.

In the population-based cohort, COL1α1 levels in plasma were significantly lower in those who developed CAD compared to those who did not. A cox proportional hazard analysis confirmed the association between lower COL1α1 levels and CAD outcome, even after multiple adjustment. The results confirm, and extend, the previous findings by Ong *et al.* (18). However, in that explorative study, the measurements of COL1α1 were based on relative quantification, and it was therefore important to validate the results by a quantifying assay. Prior to the study by Ong *et al.* (18), there were no studies available on COL1α1 in a CAD setting, except for one study reporting that a COL1α1 gene polymorphism occurred more frequently in MI survivors, as compared to age-matched controls (23).

Similar to COL1α1, PRO-C1, a biomarker of type I collagen synthesis, was inversely associated with CAD in both unadjusted and adjusted models while C1M, a biomarker for type I collagen breakdown, did not show any association. In line with this, COL1α1 showed a strong correlation with PRO-C1 indicating that both are markers of type I collagen synthesis. COL1α1 is the α1 chain partly making up the type I collagen molecule, whilst PRO-C1 is the internal epitope in the N-terminal pro-peptide of type I collagen (PINP). Hence, they can be regarded as products from two different stages of type I collagen synthesis (9, 10). To our knowledge, no previous studies have investigated PRO-C1 in the context of CAD. On the other hand, C1M has been associated with increased risk of cardiovascular events. One previous study reported that elevated C1M levels predicted MI in a prospective cohort of postmenopausal women (n=5 450, median age 71 years) with a median follow-up time of 14 years (17). Another study with a follow-up of 6 years reported that elevated C1M levels predicted cardiovascular events and mortality in patients who had undergone carotid endarterectomy (n = 473, median age 72 years, 64% men) (16). The discrepancy in results between these previous studies and the present study as regards C1M could be due to several reasons. First, the two previous studies measured C1M in serum while we utilized plasma, a distinction that can lead to different outcomes with examples from other biomarkers such as MMPs (24). Moreover, the study populations are not comparable in terms of size and composition. For example, the study by Holm Nielsen *et al.* (16) included patients who had undergone carotid endarterectomy yielding an older cohort with high atherosclerotic burden compared to our population-based cohort.

In the present study, we also investigated the temporal pattern of COL1α1 levels in MI patients by performing serial measurements from admission up to 6 months after the acute event. COL1α1 did not show any temporal changes over time in STEMI or NSTEMI patients, neither did it show any correlation with inflammatory markers or hsTnT in the early phase. Obviously, COL1α1 levels in plasma were not associated with acute atherothrombosis, inflammation or myocardial injury. Interestingly, COL1α1 values in patients with acute MI in the APACHE cohort were found to be comparable to COL1α1 values in individuals who subsequently developed incident CAD in LSH, as shown in **Figure 2**. However, this comparison should be made with caution since it is based on two distinct cohorts.

For peer review only - http://bmjopen.bmj.com/site/about/guidelines.xhtml

Women had significantly higher levels of COL1α1 and PRO-C1 compared to men in the LSH cohort. Female sex was also the only determinant that remained independently associated with COL1α1 and PRO-C1 in a multiple regression model including baseline variables. Collagen homeostasis has been shown to be affected by estrogen (25). PRO-C1 and other bone turnover markers are known to increase in women after menopause while there is little increase in men with age (26). This may partly explain why women in our population-based cohort had higher levels of COL1α1 and PRO-C1 than men. The sex difference in COL1α1 was also observed in the APACHE cohort, however these data should be interpreted with caution due to the limited number of female subjects in APACHE.

The major limitation of our study is the limited sample size, especially as regards women. Also, our cohorts reflected the Swedish population, which may limit the generalizability of results to other populations. Furthermore, in the LSH-cohort, case-control matching was performed based only on age and sex, which might result in differences between the groups that could affect the study outcome. To address this issue, other factors known to be of major importance for CAD risk were included in adjustment models. Still, it is possible that we have residual confounding factors contributing to the association between COL1α1 and risk of CAD. A recent study showed that COL1α1 was upregulated in left ventricular heart tissue from heart failure patients and that plasma levels were increased in those who were candidates for heart transplant (27). However, the MI cohort in our study included only 16 patients with moderate or severe left ventricular dysfunction, which does not permit us to draw conclusions about the relationship between COL1α1 and heart failure.

Based on our results, it can be speculated that plasma levels of COL1α1 and PRO-C1 reflect the ability to form type I collagen in arterial tissue, thereby influencing the development and fate of atherosclerotic plaques and risk of MI. However, bones and tendons are other sources of COL1α1 in the circulation. Also, the liver may leak ECM components to the blood stream under pathological conditions (28). Whether COL1α1 itself has a causal role in the development of CAD is unclear. Interestingly, a few previous clinical studies have investigated the effect of collagen supplement therapies on atherosclerosis-related factors. These studies, although small and with short intervention periods, have shown positive effects on LDL cholesterol/HDL cholesterol ratio, toxic advanced glycation end-products, cardio-ankle vascular index and brachial-ankle pulse wave velocity (29, 30).

To summarize, we confirm that absolute concentrations of COL1α1 in plasma are independently and inversely associated with future risk of CAD, but does not seem to change in an acute coronary event setting. COL1α1 in plasma appears to reflect collagen synthesis, but not collagen degradation, inflammation or myocardial injury. Larger studies are needed to confirm the clinical usefulness of COL1α1 as a marker of coronary atherosclerosis and/or predictor of incident CAD.

For peer review only - http://bmjopen.bmj.com/site/about/guidelines.xhtml

Acknowledgments

We would like to acknowledge Bettina Jung and Sofie Madsen for their technical support. The research was supported by a grant from the County Council of Östergötland, Sweden, aimed towards scientists early in their career; Academy of Health Care, County Council of Jönköping (Futurum); and The Danish Research Foundation and Innovation Fund Denmark.

Competing interests

SHN is full-time employees at Nordic Bioscience A/S. Nordic Bioscience is a privately-owned, small–medium size enterprise (SME) partly focused on the development of biomarkers including PRO-C1 and C1M. No fees, bonuses or other benefits was rewarded for the work described in the manuscript. SHN hold stocks in Nordic Bioscience A/S.

Author contributions

Filip Hammaréus wrote the manuscript; Filip Hammaréus, Anna Lundberg, Lena Jonasson, Margareta Kristenson, Kwok Leung Ong, Eva Swahn and Joakim Alfredsson participated in study design; Filip Hammaréus, Rosanna WS Chung and Anna Kristina Lundberg prepared plasma samples and performed the Luminex-assay on COL1α1; Signe Holm Nielsen measured C1M and PRO-C1 levels in the LSH cohort; Filip Hammaréus, Karin Festin, Lennart Nilsson and Joakim Alfredsson participated in data analysis; All authors participated in data interpretation and critical revision of the manuscript.

Data availability statement

Data are available on reasonable request.

Funding source

The LSH study was funded by the Swedish Research Council (2004−1881) and the Swedish Heart and Lung Foundation (2004053). The APACHE study received financial support from ALF Region Östergotland (LIO 131 471). The research was supported by the County Council of Östergötland, specifically with a grant aimed towards scientists early in their career (RÖ-910951); Academy of Health Care, County Council of Jönköping (N/A); and The Danish Research Foundation (N/A) and Innovation Fund Denmark (N/A). Further, we acknowledge the support from Linköping university. The funding sources had no role in study design, execution, analysis, interpretation of results or decision to submit.

Ethical considerations

As previously described, the LSH and APACHE studies have been approved by The Regional Ethical Review Board in Linköping (Dnr 02–0324 and M45-08 respectively). All study participants have given written informed consent.

For peer review only - http://bmjopen.bmj.com/site/about/guidelines.xhtml

References

1. Herrington W, Lacey B, Sherliker P, Armitage J, Lewington S. Epidemiology of Atherosclerosis and the Potential to Reduce the Global Burden of Atherothrombotic Disease. Circ Res. 2016;118(4):535-46.
2. Bentzon JF, Otsuka F, Virmani R, Falk E. Mechanisms of plaque formation and rupture. Circ Res. 2014;114(12):1852-66.
3. Rekhter MD. Collagen synthesis in atherosclerosis: too much and not enough. Cardiovasc Res. 1999;41(2):376-84.
4. Galis ZS, Khatri JJ. Matrix metalloproteinases in vascular remodeling and atherogenesis: the good, the bad, and the ugly. Circ Res. 2002;90(3):251-62.
5. Galis ZS, Sukhova GK, Lark MW, Libby P. Increased expression of matrix metalloproteinases and matrix degrading activity in vulnerable regions of human atherosclerotic plaques. J Clin Invest. 1994;94(6):2493-503.
6. Gough PJ, Gomez IG, Wille PT, Raines EW. Macrophage expression of active MMP-9 induces acute plaque disruption in apoE-deficient mice. J Clin Invest. 2006;116(1):59-69.
7. Blankenberg S, Rupprecht HJ, Poirier O, Bickel C, Smieja M, Hafner G, et al. Plasma concentrations and genetic variation of matrix metalloproteinase 9 and prognosis of patients with cardiovascular disease. Circulation. 2003;107(12):1579-85.
8. Garvin P, Jonasson L, Nilsson L, Falk M, Kristenson M. Plasma Matrix Metalloproteinase-9 Levels Predict First-Time Coronary Heart Disease: An 8-Year Follow-Up of a Community-Based Middle Aged Population. PLoS One. 2015;10(9):e0138290.
9. Ricard-Blum S. The collagen family. Cold Spring Harb Perspect Biol. 2011;3(1):a004978.
10. Leeming DJ, Larsen DV, Zhang C, Hi Y, Veidal SS, Nielsen RH, et al. Enzyme-linked immunosorbent serum assays (ELISAs) for rat and human N-terminal pro-peptide of collagen type I (PINP)--assessment of corresponding epitopes. Clin Biochem. 2010;43(15):1249-56.
11. Leeming D, He Y, Veidal S, Nguyen Q, Larsen D, Koizumi M, et al. A novel marker for assessment of liver matrix remodeling: an enzyme-linked immunosorbent assay (ELISA) detecting a MMP generated type I collagen neo-epitope (C1M). Biomarkers. 2011;16(7):616-28.
12. Murata K, Motayama T, Kotake C. Collagen types in various layers of the human aorta and their changes with the atherosclerotic process. Atherosclerosis. 1986;60(3):251-62.
13. Shami A, Gonçalves I, Hultgårdh-Nilsson A. Collagen and related extracellular matrix proteins in atherosclerotic plaque development. Curr Opin Lipidol. 2014;25(5):394-9.
14. Edsfeldt A, Gonçalves I, Grufman H, Nitulescu M, Dunér P, Bengtsson E, et al. Impaired fibrous repair: a possible contributor to atherosclerotic plaque vulnerability in patients with type II diabetes. Arterioscler Thromb Vasc Biol. 2014;34(9):2143-50.
15. Wang J, Geng YJ, Guo B, Klima T, Lal BN, Willerson JT, et al. Near-infrared spectroscopic characterization of human advanced atherosclerotic plaques. J Am Coll Cardiol. 2002;39(8):1305-13.
16. Holm Nielsen S, Tengryd C, Edsfeldt A, Brix S, Genovese F, Bengtsson E, et al. A biomarker of collagen type I degradation is associated with cardiovascular events and mortality in patients with atherosclerosis. J Intern Med. 2019;285(1):118-23.
17. Bertelsen DM, Neergaard JS, Bager CL, Nielsen SH, Secher NH, Svendsen JH, et al. Matrix Metalloproteinase Mediated Type I Collagen Degradation is an Independent

For peer review only - http://bmjopen.bmj.com/site/about/guidelines.xhtml

Predictor of Increased Risk of Acute Myocardial Infarction in Postmenopausal Women. Sci Rep. 2018;8(1):5371.
18. Ong KL, Chung RWS, Hui N, Festin K, Lundberg AK, Rye KA, et al. Usefulness of Certain Protein Biomarkers for Prediction of Coronary Heart Disease. Am J Cardiol. 2020;125(4):542-8.
19. Alfredsson J, Lindahl TL, Gustafsson KM, Janzon M, Jonasson L, Logander E, et al. Large early variation of residual platelet reactivity in Acute Coronary Syndrome patients treated with clopidogrel: results from Assessing Platelet Activity in Coronary Heart Disease (APACHE). Thromb Res. 2015;136(2):335-40.
20. Thygesen K, Alpert JS, White HD, Joint ESCAAHAWHFTFftRoMI. Universal definition of myocardial infarction. J Am Coll Cardiol. 2007;50(22):2173-95.
21. Lang RM, Bierig M, Devereux RB, Flachskampf FA, Foster E, Pellikka PA, et al. Recommendations for chamber quantification. Eur J Echocardiogr. 2006;7(2):79-108.
22. Zhang Y, Li X, Di YP. Fast and Efficient Measurement of Clinical and Biological Samples Using Immunoassay-Based Multiplexing Systems. Methods Mol Biol. 2020;2102:129-47.
23. Speer G, Szenthe P, Kosa JP, Tabak AG, Folhoffer A, Fuszek P, et al. Myocardial infarction is associated with Spl binding site polymorphism of collagen type 1A1 gene. Acta Cardiol. 2006;61(3):321-5.
24. Mannello F. Serum or plasma samples? The "Cinderella" role of blood collection procedures: preanalytical methodological issues influence the release and activity of circulating matrix metalloproteinases and their tissue inhibitors, hampering diagnostic trueness and leading to misinterpretation. Arterioscler Thromb Vasc Biol. 2008;28(4):611-4.
25. McRobb LS, McGrath KCY, Tsatralis T, Liong EC, Tan JTM, Hughes G, et al. Estrogen Receptor Control of Atherosclerotic Calcification and Smooth Muscle Cell Osteogenic Differentiation. Arterioscler Thromb Vasc Biol. 2017;37(6):1127-37.
26. Eastell R, Pigott T, Gossiel F, Naylor KE, Walsh JS, Peel NFA. DIAGNOSIS OF ENDOCRINE DISEASE: Bone turnover markers: are they clinically useful? Eur J Endocrinol. 2018;178(1):R19-r31.
27. Hua X, Wang YY, Jia P, Xiong Q, Hu Y, Chang Y, et al. Multi-level transcriptome sequencing identifies COL1A1 as a candidate marker in human heart failure progression. BMC Med. 2020;18(1):2.
28. Nallagangula KS, Nagaraj SK, Venkataswamy L, Chandrappa M. Liver fibrosis: a compilation on the biomarkers status and their significance during disease progression. Future Sci OA. 2018;4(1):FSO250.
29. Tomosugi N, Yamamoto S, Takeuchi M, Yonekura H, Ishigaki Y, Numata N, et al. Effect of Collagen Tripeptide on Atherosclerosis in Healthy Humans. J Atheroscler Thromb. 2017;24(5):530-8.
30. Igase M, Kohara K, Okada Y, Ochi M, Igase K, Inoue N, et al. A double-blind, placebo-controlled, randomised clinical study of the effect of pork collagen peptide supplementation on atherosclerosis in healthy older individuals. Biosci Biotechnol Biochem. 2018;82(5):893-5.

For peer review only - http://bmjopen.bmj.com/site/about/guidelines.xhtml

Figure legends

Figure 1. Kaplan Meier survival curves showing cumulative survival in terms of primary outcome (MI or invasive coronary intervention) between participants with high versus low (above or under median) COL1α1 or PRO-C1 values in the LSH cohort (COL1α1 = collagen type I α1 chain; Cum = cumulative)

Figure 2. Box plots showing COL1α1 plasma levels (pg/mL) measured by the Luminex® Assay among participants in LSH and APACHE. *P*-value based on Mann-Whitney U test (APACHE = Assessing Platelet Activity in Coronary Heart Disease; COL1α1 = collagen type I α1 chain; LSH = Lifeconditions, Stress and Health study)

*At admission

Supplementary figure 1. Scatter plot showing COL1α1 plasma levels in LSH participants based on Luminex-analysis (y-axis, $\log_2$) versus PEA-analysis (x-axis) along with linear regression line (COL1α1 = collagen type I α1 chain; Log2 = logarithmized with the base 2; NPX = normalized protein expression)

Supplementary figure 2. Box plots showing COL1α1 plasma levels (pg/mL) measured by the Luminex® Assay in LSH and APACHE cohorts split on sex. *P*-values based upon Mann-Whitney U tests (APACHE = Assessing Platelet Activity in Coronary Heart Disease; COL1α1 = collagen type I α1 chain; LSH = Lifeconditions, Stress and Health study)

*At admission

** 6 months after admission

For peer review only

Tables

Table 1. Baseline characteristics of cases and controls in the LSH cohort

Characteristics	Controls (n=184)	Cases (n=86)	*p*
Age, years	60.6 (6.4)	60.6 (6.6)	0.84
Women, n (%)	66 (36)	31 (36)	>0.9
Smokers, n (%)	19 (11)	24 (30)	<0.001
BMI, kg/m^2	26.8 (3.6)	27.8 (3.8)	0.048
Systolic blood pressure, mmHg	135 (18)	146 (24)	<0.001
Antihypertensive medication, n (%)	32 (17)	30 (35)	0.002
Lipid-lowering medication, n (%)	9 (4.9)	11 (13)	0.027
Diabetes, n (%)	14 (7.6)	8 (9.3)	0.64
Angina pectoris, n (%)	4 (2.2)	12 (14)	<0.001
Serious chronic illness, n (%)	13 (7.1)	9 (11)	0.34
HDL-cholesterol, mmol/L [a]	1.50 (0.50)	1.40 (0.42)	0.001
LDL-cholesterol, mmol/L	3.54 (0.82)	3.64 (0.90)	0.39
Non-HDL cholesterol, mmol/L	4.11 (0.89)	4.36 (0.91)	0.036
Triglycerides, mmol/L[a]	1.10 (0.66)	1.40 (0.92)	<0.001
CRP, mg/L [a]	0.835 (1.7)	1.56 (2.4)	0.01
IL-6, pg/mL [a]	0.560 (2.25)	1.68 (2.5)	0.097
MMP-9, ng/mL [a]	30.7 (32)	39.5 (31)	0.036

BMI = body mass index; HDL = high density lipoprotein; LDL = low density lipoprotein; CRP = C-reactive protein; IL-6 = interleukin-6; MMP-9 = matrix metalloproteinase-9

Data expressed as mean (SD) or median (IQR)[a] if data do not possess distribution of normality. *P*-values from comparing controls and cases in terms of characteristics.

For peer review only

Table 2. Circulating levels of COL1α1, PRO-C1, C1M and their association with incident coronary artery disease at 13-year follow-up in the LSH cohort

Biomarker	Median (IQR) level				Unadjusted model		Adjusted model†	
	Control (n=184*)	Case (n=86*)	Difference	*p*	HR (95% CI)	*p*	HR (95% CI)	*p*
COL1α1 (pg/mL)	4430 (2900)	3810 (3400)	16%	0.002	0.69 (0.56-0.87)	0.001	0.55 (0.41-0.75)	<0.001
PRO-C1 (ng/mL)	77.6 (43)	59.7 (39)	30%	<0.001	0.62 (0.47-0.82)	0.001	0.61 (0.43-0.86)	0.005
C1M (ng/mL)	22.0 (16)	23.9 (19)	8.6%	*ns*	1.57 (0.568-4.34)	NS	0.92 (0.29-3.37)	*ns*

COL1α1 = collagen type I α1 chain; PRO-C1 = N-terminal pro-peptide of type I collagen; C1M = MMP-mediated degradation of type I collagen; HR = hazard ratio; *ns* = non-significant

Association expressed as hazard ratios per one unit increase in log2-tranformed unit from Cox proportional hazard analysis

*For pro-C1 and C1M, n=172 and n=83 for controls and cases respectively
† Covariates being adjusted for were sex, age, systolic blood pressure, smoking, body mass index, triglycerides, high-density lipoprotein (HDL) cholesterol, non-HDL cholesterol, C-reactive protein (CRP), use of anti-hypertensive and lipid-lowering medication, and presence of chronic disease

For peer review only

Table 3. Baseline characteristics of 125 patients with acute MI in the APACHE cohort

Characteristics	Total (n=125)	STEMI (n=73)	NSTEMI (n=52)	*p*
Age, years	66.0 (12)	65.9 (13)	66.1 (11)	0.86
Women, n (%)	37 (30)	25 (34)	12 (23)	0.18
Smokers, n (%)	34 (27)	25 (34)	9 (17)	0.037
BMI, kg/m^2 [a]	26.3 (4.4)	26.2 (4.2)	26.3 (4.1)	0.59
Systolic blood pressure, mmHg	148 (28)	141 (26)	159 (28)	0.001
Diastolic blood pressure, mmHg	83 (17)	81 (15)	86 (20)	0.14
Lipid-lowering medication, n (%) [b]	31 (25)	13 (18)	18 (35)	0.033
Diabetes, n (%)	16 (13)	7 (9.5)	9 (17)	0.18
LV-function, normal/mildly reduced, n (%)	109 (87)	60 (82)	49 (94)	<0.001
hsTnT (at 6-8 h), ng/mL [a]	9.12 (91)	7.90 (130)	17.5 (71)	0.68
HDL-cholesterol, mmol/L [a]	1.10 (0.37)	1.10 (0.39)	1.10 (0.45)	0.59
LDL-cholesterol, mmol/L	2.84 (0.90)	2.94 (0.89)	2.67 (0.89)	0.12
Non-HDL cholesterol, mmol/L [a]	3.49 (1.3)	3.50 (1.3)	3.40 (1.5)	0.51
Triglycerides, mmol/L [a]	1.45 (0.87)	1.40 (0.75)	1.50 (1.1)	0.50
CRP, mg/L [a]	3.66 (6.0)	4.44 (5.3)	3.16 (7.0)	0.34
IL-6, pg/mL [a]	3.58 (5.0)	4.36 (5.8)	2.64 (3.0)	0.005
MMP-9, ng/mL [a]	263 (210)	253 (170)	310 (230)	0.62
COL1α1, pg/mL [a]	3590 (3200)	3670 (3900)	3580 (2400)	0.31

BMI = body mass index; hsTnT = high sensitivity Troponin T; HDL = high density lipoprotein; CRP = C-reactive protein; IL-6 = interleukin-6; MMP-9 = matrix metalloproteinase-9; COL1α1 = collagen type I α1 chain.

Statistics expressed as mean (SD or median (IQR)[a] if data do not possess distribution of normality. P-values from comparing STEMI and NSTEMI patients in terms of characteristics. Laboratory variables represent levels at admission (if not otherwise indicated).

[b] Defined as statin use at arrival to cardiology department

For peer review only - http://bmjopen.bmj.com/site/about/guidelines.xhtml

For peer review only

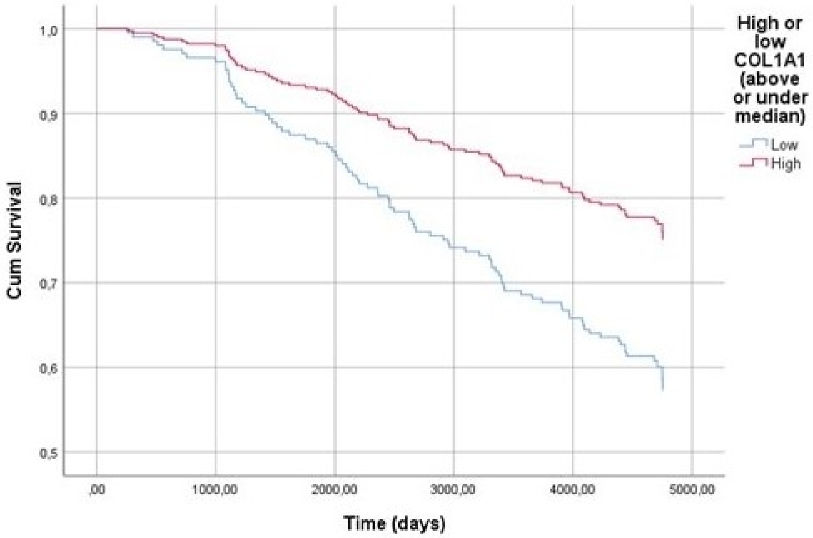

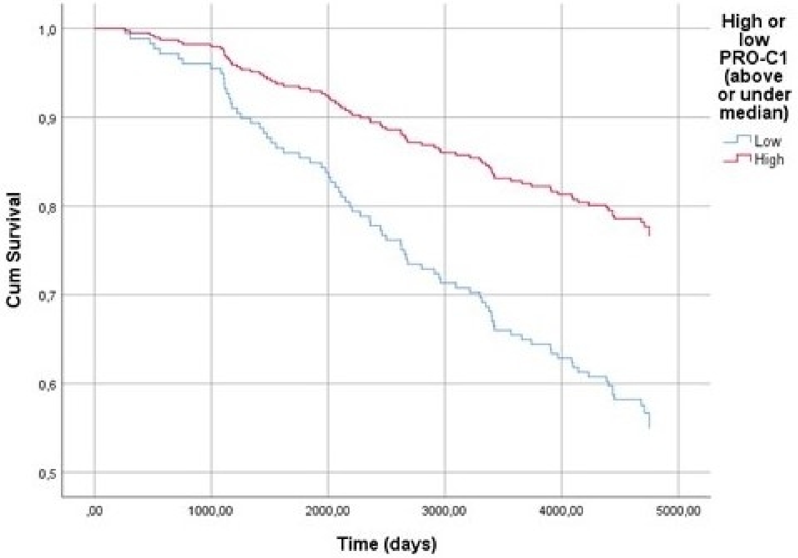

Kaplan Meier survival curves showing cumulative survival in terms of primary outcome (MI or invasive coronary intervention) between participants with high versus low (above or under median) COL1α1 or PRO-C1 values in the LSH cohort (COL1α1 = collagen type I α1 chain; Cum = cumulative)

137x189mm (96 x 96 DPI)

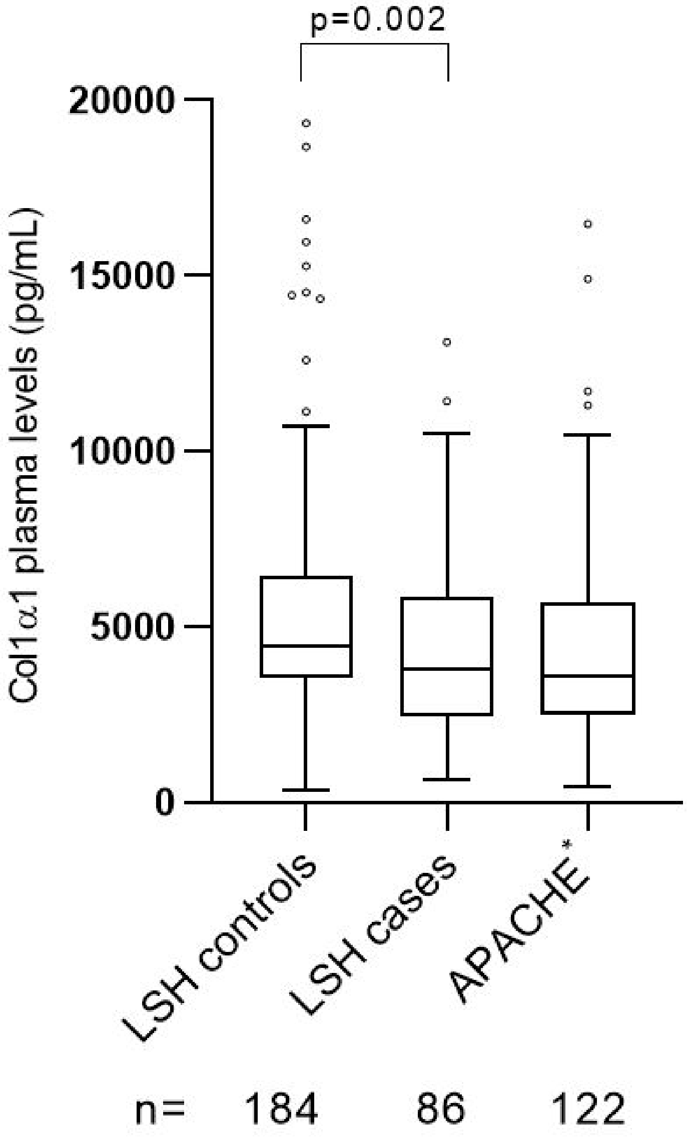

Figure 2. Box plots showing COL1α1 plasma levels (pg/mL) measured by the Luminex® Assay among participants in LSH and APACHE. P-value based on Mann-Whitney U test (APACHE = Assessing Platelet Activity in Coronary Heart Disease; COL1α1 = collagen type I α1 chain; LSH = Lifeconditions, Stress and Health study)
*At admission

73x120mm (144 x 144 DPI)

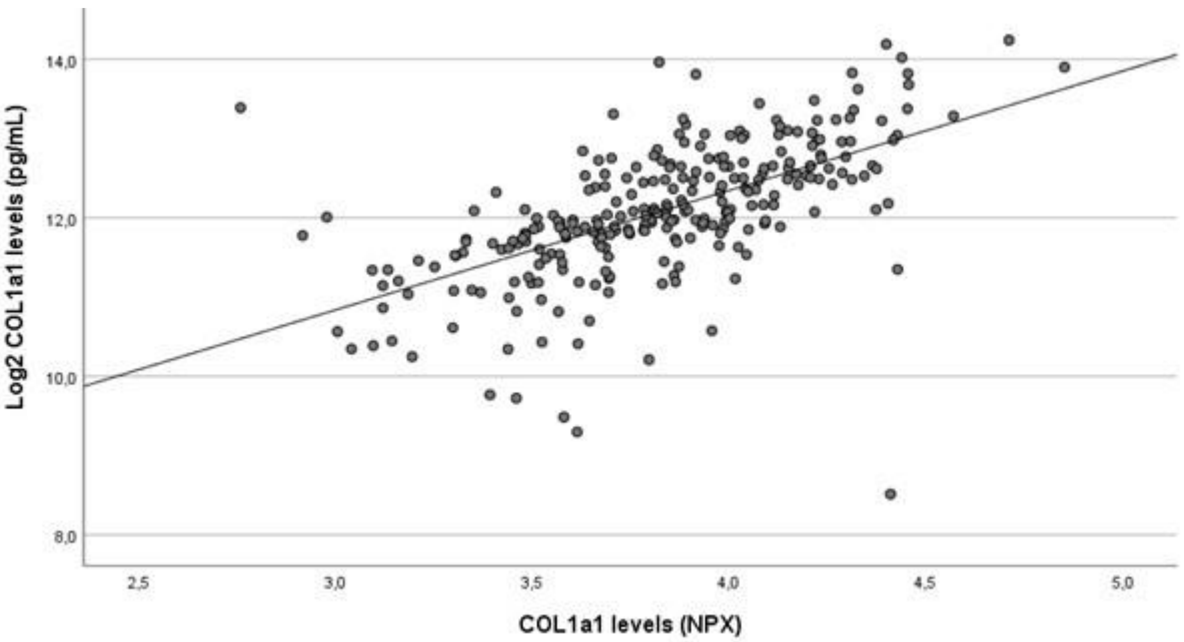

Supplementary figure 1. Scatter plot showing COL1α1 plasma levels in LSH participants based on Luminex-analysis (y-axis, log_2) versus PEA-analysis (x-axis) along with linear regression line (COL1α1 = collagen type I α1 chain; Log2 = logarithmized with the base 2; NPX = normalized protein expression)

peer review only

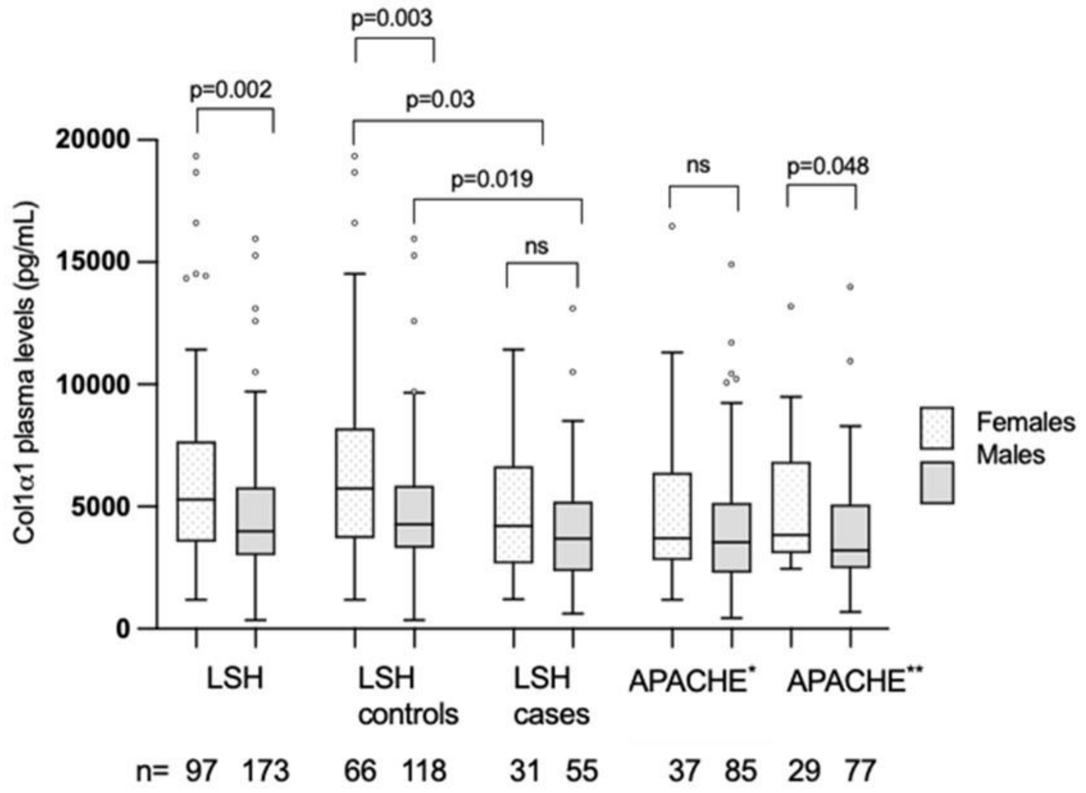

Supplementary figure 2. Box plots showing COL1α1 plasma levels (pg/mL) measured by the Luminex® Assay in LSH and APACHE cohorts split on sex. *P*-values based upon Mann-Whitney U tests (APACHE = Assessing Platelet Activity in Coronary Heart Disease; COL1α1 = collagen type I α1 chain; LSH = Lifeconditions, Stress and Health study)

*At admission

** 6 months after admission

For peer review only

Supplementary table 1. Correlations between COL1α1, PRO-C1, C1M, demographic, clinical and laboratory variables in the LSH cohort expressed as correlation coefficients (*r*) and *p*-values

Variable	COL1α1		PRO-C1		C1M	
	r	*p*	*r*	*p*	*r*	*p*
Age	-0.01	*ns*	-0.08	*ns*	0.13	0.035
Sex†	0.19	0.002	0.21	0.001	0.13	0.045
BMI	-0.03	*ns*	-0.16	0.015	0.15	0.015
Diabetes†	-0.15	0.014	-0.09	*ns*	0.13	0.04
Angina pectoris†	-0.18	0.003	-0.17	0.007	0.08	*ns*
HDL cholesterol	0.13	0.029	0.19	0.003	-0.02	*ns*
Non-HDL cholesterol	0.07	*ns*	-0.04	*ns*	0.13	0.041
Triglycerides	-0.08	*ns*	-0.17	0.007	0.14	0.033
CRP	0.06	*ns*	0.01	*ns*	0.30	<0.001
IL-6	-0.05	*ns*	-0.16	0.017	0.21	0.001
MMP-9	-0.10	*ns*	-0.20	0.002	0.07	*ns*
Pro-C1	0.73	<0.001	-	-	-0.03	*ns*
C1M	<0.01	*ns*	-0.04	*ns*	-	-

COL1α1 = collagen type I α1 chain; BMI = body mass index; HDL = high density lipoprotein; CRP = C-reactive protein; IL-6 = interleukin-6; MMP-9 = matrix metalloproteinase-9; *ns* = non-significant
Correlations calculated with Spearman analysis. $p < 0.05$ considered statistically significant
† Male coded as 1 and female as 2, diabetes coded as 2 and not having diabetes coded as 1, angina pectoris coded as 2 and not having angina pectoris coded as 1

For peer review only

STROBE Statement—checklist of items that should be included in reports of observational studies

All references to pages and lines are based on the manuscript version where tracked changes are remaining.

	Item No	Recommendation
**Title and abstract**	1	(*a*) Indicate the study's design with a commonly used term in the title or the abstract Page 1, line 1-3 (added prospective to enhance the description of the 2nd cohort) + page 2 line 53-55
		(*b*) Provide in the abstract an informative and balanced summary of what was done and what was found Page 2
**Introduction**		
Background/rationale	2	Explain the scientific background and rationale for the investigation being reported Page 4 line 108-143.
Objectives	3	State specific objectives, including any prespecified hypotheses Page 4-5 line 145-153
**Methods**		
Study design	4	Present key elements of study design early in the paper Page 6
Setting	5	Describe the setting, locations, and relevant dates, including periods of recruitment, exposure, follow-up, and data collection Page 5 + page 6 line 209-210
Participants	6	(*a*) *Cohort study*—Give the eligibility criteria, and the sources and methods of selection of participants. Describe methods of follow-up Page 6 line 160-163 + line 171-175 + line 186-191 + line 197-198<br>*Case-control study*—Give the eligibility criteria, and the sources and methods of case ascertainment and control selection. Give the rationale for the choice of cases and controls Page 6 line 160-163 + line 171-175 + line 177-182 + page 5 line 135-142<br>*Cross-sectional study*—Give the eligibility criteria, and the sources and methods of selection of participants Not applicable
		(*b*) *Cohort study*—For matched studies, give matching criteria and number of exposed and unexposed Not applicable<br>*Case-control study*—For matched studies, give matching criteria and the number of controls per case Page 6 line 179-181
Variables	7	Clearly define all outcomes, exposures, predictors, potential confounders, and effect modifiers. Give diagnostic criteria, if applicable Page 6 line 163-181 + line 191-203
Data sources/ measurement	8*	For each variable of interest, give sources of data and details of methods of assessment (measurement). Describe comparability of assessment methods if there is more than one group Page 7 line 205-245. Same measurement method was used for cases and controls.
Bias	9	Describe any efforts to address potential sources of bias Page 8 line 259-265 + page 7 line 223-225
Study size	10	Explain how the study size was arrived at Page 6 line 160-162 + line 177-181 + line 185-189. Two already existing cohorts.
Quantitative variables	11	Explain how quantitative variables were handled in the analyses. If applicable, describe which groupings were chosen and why Page 7 line 248-251 + page 8 line 265-266
Statistical methods	12	(*a*) Describe all statistical methods, including those used to control for confounding Page 7-8, line 247-270
		(*b*) Describe any methods used to examine subgroups and interactions Table 1 (page 18) + Table 3 (page 20) + page 8 line 254-257

For peer review only - http://bmjopen.bmj.com/site/about/guidelines.xhtml

	(*c*) Explain how missing data were addressed Page 6 line 175-177 + page 7 line 222-223 + line 243-245 + page 7-8 line 251-252
	(*d*) *Cohort study*—If applicable, explain how loss to follow-up was addressed. Not applicable, no cases/controls were lost to follow-up.<br>*Case-control study*—If applicable, explain how matching of cases and controls was addressed. Page 6 line 179-182<br>*Cross-sectional study*—If applicable, describe analytical methods taking account of sampling strategy Not applicable
	(*e*) Describe any sensitivity analyses Page 7 line 223-225

Continued on next page

For peer review only

For peer review only - http://bmjopen.bmj.com/site/about/guidelines.xhtml

**Results**		
Participants	13*	(a) Report numbers of individuals at each stage of study—eg numbers potentially eligible, examined for eligibility, confirmed eligible, included in the study, completing follow-up, and analysed Page 6 line 160-163 + line 175-181. Number of individuals eligible and excluded from APACHE not available.
		(b) Give reasons for non-participation at each stage Page 6 line 162-163 + line 175-177. Not available for APACHE.
		(c) Consider use of a flow diagram Not applicable given two cohorts which are described previously.
Descriptive data	14*	(a) Give characteristics of study participants (eg demographic, clinical, social) and information on exposures and potential confounders Page 9-10 results section + Table 1-3 (page 18-20)
		(b) Indicate number of participants with missing data for each variable of interest Page 6 line 169-171 + line 197-201 + Page 7 line 222-223 + line 243-245 + page 7-8 line 251-252
		(c) *Cohort study*—Summarise follow-up time (eg, average and total amount) Page 6 line 171-172 + 178-179
Outcome data	15*	*Cohort study*—Report numbers of outcome events or summary measures over time Page 6 line 177-179
		Case-control study—Report numbers in each exposure category, or summary measures of exposure Not applicable (nested case-control design)
		Cross-sectional study—Report numbers of outcome events or summary measures Not applicable
Main results	16	(*a*) Give unadjusted estimates and, if applicable, confounder-adjusted estimates and their precision (eg, 95% confidence interval). Make clear which confounders were adjusted for and why they were included Page 8 line 261-265 + page 9 line 294-298 + 303-311 + Table 2 (page 19)
		(*b*) Report category boundaries when continuous variables were categorized Page 9 line 298-301 + 308-311 + page 6 line 195-197
		(*c*) If relevant, consider translating estimates of relative risk into absolute risk for a meaningful time-period. Not applicable.
Other analyses	17	Report other analyses done—eg analyses of subgroups and interactions, and sensitivity analyses Page 9-10 results section, table 1 and 3 (page 18,20)
**Discussion**		
Key results	18	Summarise key results with reference to study objectives Page 11 line 350-357
Limitations	19	Discuss limitations of the study, taking into account sources of potential bias or imprecision. Discuss both direction and magnitude of any potential bias Page 12 line 414-425
Interpretation	20	Give a cautious overall interpretation of results considering objectives, limitations, multiplicity of analyses, results from similar studies, and other relevant evidence Page 12 line 427-436 + previous paragraphs in discussion
Generalisability	21	Discuss the generalisability (external validity) of the study results Page 12 line 414-416
**Other information**		
Funding	22	Give the source of funding and the role of the funders for the present study and, if applicable, for the original study on which the present article is based Page 1 line 28-35

*Give information separately for cases and controls in case-control studies and, if applicable, for exposed and unexposed groups in cohort and cross-sectional studies.

For peer review only

Note: An Explanation and Elaboration article discusses each checklist item and gives methodological background and published examples of transparent reporting. The STROBE checklist is best used in conjunction with this article (freely available on the Web sites of PLoS Medicine at http://www.plosmedicine.org/, Annals of Internal Medicine at http://www.annals.org/, and Epidemiology at http://www.epidem.com/). Information on the STROBE Initiative is available at www.strobe-statement.org.

For peer review only
